# Impact of tractogram filtering and graph creation for structural connectomics in subjects with Parkinson's disease

**DOI:** 10.3389/fnhum.2026.1769103

**Published:** 2026-04-23

**Authors:** Fabian Leander Sinzinger, Sanna Persson, Marvin Köpff, Joana B. Pereira, Rodrigo Moreno

**Affiliations:** 1 Division of Biomedical Imaging, Department of Biomedical Engineering and Health Systems, KTH Royal Institute of Technology, Stockholm, Sweden; 2 Department of Clinical Neuroscience, Karolinska Institutet, Stockholm, Sweden; 3 Department of Neurobiology, Care Sciences and Society, Karolinska Institutet, Stockholm, Sweden

**Keywords:** diffusion MRI, graph creation, Parkinson's disease, structural connectomics, tractogram filtering

## Abstract

**Introduction:**

Structural connectomics derives subject-specific brain connectivity from diffusion-weighted MRI and has potential as a biomarker for clinical Parkinson's disease (PD) detection.

**Method:**

In this study, we applied probabilistic tractography (iFOD2) to derive different types of connectomes and analyzed group discriminability between PD patients and healthy controls from the Parkinson's Progression Markers Initiative (PPMI) dataset (n = 233). Particular emphasis was placed on the streamline filtering stage with SIFT2 and the comparison of different connectivity metrics, including streamline count, fractional anisotropy (FA), axial diffusivity (AD), mean diffusivity (MD), and radial diffusivity (RD). We performed a three-level analysis comprising (1) connection-level statistical analysis, (2) graph theory measures at the node and whole-brain levels, and (3) classification using support vector machines (SVM) and graph neural networks.

**Results:**

We did not find any statistical difference at any level after correction for multiple comparisons. Also, the classifiers performed poorly with AUC values close to chance levels. However, we found differences between filtered and unfiltered tractograms at the node level.

**Discussion:**

Our findings suggest that structural connectivity analyses for PD are highly sensitive to specific pipeline configurations and fine-tuning.

## 1 Introduction

Parkinson's disease (PD) is a neurodegenerative disease impacting the motor and non-motor functions of affected patients. There is substantial evidence linking the presence and progression of PD with structural changes in the brain anatomy (Pieperhoff et al., 2022; Park et al., 2022). In particular, alterations in white matter (WM) integrity tend to occur prior to cortical thinning both for PD and dementia (Rektor et al., 2018; Niu et al., 2025; Maier-Hein et al., 2015) and may hold potential as a biomarker for early diagnosis. Moreover, such alterations of the WM integrity modify the structural connectivity between individual brain regions. A common approach to reconstructing structural connectivity using tractography. Tractography methods aim to reconstruct the underlying neural fiber pathways from diffusion magnetic resonance imaging (dMRI) data (Frau-Pascual et al., 2019). The output of tractography is a set of streamlines, usually referred to as a tractogram, which represents the geometry of fiber bundles in the white matter (WM). Structural connectomics uses tractograms to assess connectivity patterns between regions of gray matter (GM), obtaining insights into how neurodegeneration affects the connectivity between pairwise regions.

High-quality tractograms are necessary for performing meaningful analysis in structural connectomics. Unfortunately, a current issue is that even state-of-the-art tractography methods can produce tractograms that suffer from low specificity, despite their high sensitivity (Maier-Hein et al., 2017; Daducci et al., 2016; Schilling et al., 2019). There are two main strategies to overcome this problem: either to propose more capable tractography methods or to filter out anatomically implausible streamlines from the tractogram as a postprocessing step. The latter strategy is usually referred to as tractogram filtering (Jörgens et al., 2021).

One of the most challenging aspects of current tractography is that ground truth data cannot be obtained in vivo. Although synthetic data (Girard et al., 2023), and ex vivo imaging (Maffei et al., 2022) can be used to assess the quality of tractograms, it remains to be determined whether these results can be extrapolated to in vivo scenarios, especially when dMRI data are acquired in clinical settings (Dell'Acqua et al., 2025, Ch. 25–27).

In this study, we investigate the structural connectivity differences between Parkinson's disease patients (PD) and healthy controls (HC). The design of our method and analysis partially builds on prior work by Köpff (2020), with adaptations to the present study. Our analysis centers around the following key questions:

1. Using the edges, can we identify statistically significant group differences in brain region pairs using a standardized structural connectivity pipeline?
2. Using the nodes, are there group-related changes in graph-theory measures?
3. Using the whole connectome, can a classification model tailored to this problem effectively discriminate between the two groups using connectivity maps as inputs?
4. How does tractogram filtering impact the validity and discriminative power of the observed differences between brain regions?

To address these questions, the paper is organized as follows. Section 2 outlines the connectivity pipeline, detailing the design choices and the rationale behind them. Results are presented in Section 3 and discussed in Section 4. Both sections are divided into three subsections, each corresponding to a specific analysis method. The first subsection presents statistical group comparisons at the brain network edge level to address the first research question. The second subsection discusses the results of the graph metrics evaluation related to the second research question. The third subsection compares and evaluates two classification models: a support vector machine classifier and an attention-based graph network classifier to answer the third research question. The analyses are performed with filtered and non-filtered tractograms to answer the fourth research question.

## 2 Methods

### 2.1 Datasets

#### 2.1.1 Parkinson's Progression Markers Initiative

The Parkinson's Progression Markers Initiative (PPMI) is a multi-site collaborative initiative to acquire a publicly available dataset on medical data from Parkinson patients (Marek et al., 2011).

Among the PD subjects available in the PPMI database, a subset was selected for this study according to the subject selection criteria aligned with those used in the study by Pereira et al. (2014). We included subjects who met standard diagnostic criteria for PD at baseline, had received their diagnosis within two years, had not yet started treatment for PD, and showed a significant dopamine transporter deficit, as confirmed via dopamine transporter imaging (DAT). For the control group, inclusion criteria included the absence of neurological dysfunction, no first-degree family history of PD, and a Montreal Cognitive Assessment (MoCA) score of 26 or higher. PD subjects were excluded if they had other significant neurological diagnoses or if they were already receiving PD treatment. Controls were excluded if they had a history of any neurological or cognitive disorders, first-degree relatives with PD, or abnormal DAT imaging results. After applying these criteria and removing cases with missing or corrupted data, a total of *n* = 233 subjects were selected for inclusion in this study. Demographic characteristics for the included subjects are summarized in Table 1. Notice that there are more PD males than females in the cohort, consistent with the fact that PD disproportionately affects more men (Hirsch et al., 2016).

**Table 1:** PPMI: Selected subjects demographics.

	Sex	Number	Age (Mean ±STD)
Control	F	24	58.79 ± 8.56
	M	43	61.81 ± 11.97
PD	F	61	60.12 ± 8.99
	M	105	61.68 ± 9.69

### 2.2 Structural connectivity pipeline

**Figure 1:**
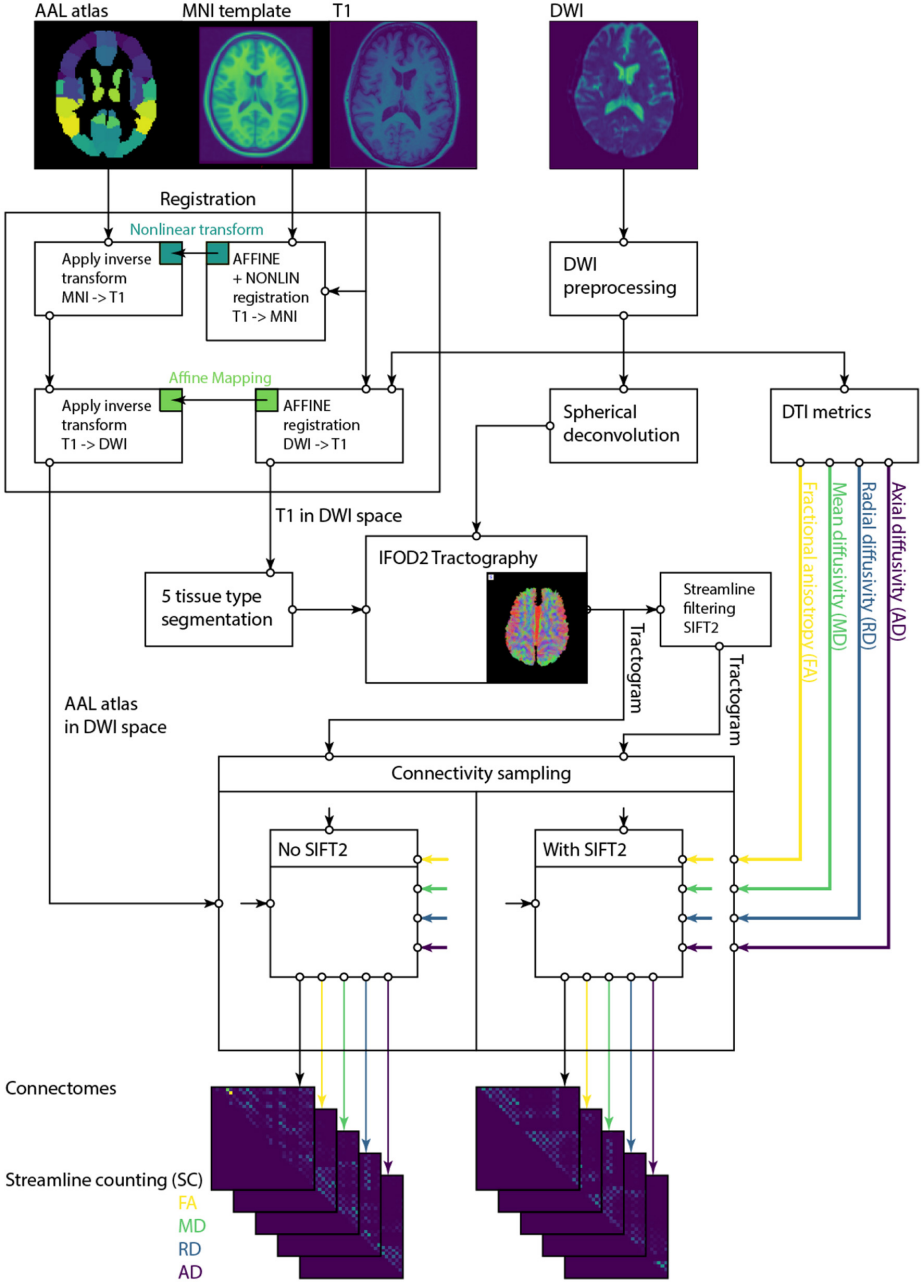
Connectivity pipeline: diffusion-weighted images are preprocessed and then converted into fiber orientation distribution images (FOD) by spherical deconvolution. Spherical deconvolution is performed using the averaged tissue-specific response function from healthy controls. Then, whole-brain (global) tractograms are tracked and filtered using SIFT2. Finally, connectomes are extracted from filtered and unfiltered tractograms weighted by streamline count (SC), fractional anisotropy (FA), mean diffusivity (MD), radial diffusivity (RD), and axial diffusivity (AD), respectively.

The structural connectivity pipeline used in this study is largely based on widely used tools in the community, particularly the work by Tahedl et al. (2025), Tournier et al. (2019), Glasser et al. (2013), Bergamino et al. (2023), and Cui et al. (2023). The primary purpose of the pipeline is to extract subject-specific brain connectivity maps (connectomes) from the diffusion-weighted images (DWIs) included in the datasets from the previous section. Connectomes assign pairwise connectivity measures to brain regions parcellated according to the automated anatomical labeling atlas (AAL atlas version 1) (Tzourio-Mazoyer et al., 2002). We excluded the cerebellar regions from our analysis, which leaves 90 cortical and subcortical regions. We also utilize T1w images from the respective subjects to automatically distinguish between different tissue types (white matter: WM, gray matter: GM, and cerebrospinal fluid: CSF) within the DWI images. An overview of the individual components of the pipeline is provided in Figure 1. The reason we describe this pipeline in such detail is that the design and parameter choices of the individual stages have downstream effects that directly and indirectly impact the results, and these should be reported for reproducibility. This variability has been shown in tractography challenges (Maier-Hein et al., 2017; Schilling et al., 2021).

#### 2.2.1 DWI pre-processing

Based on DWI acquisitions from PPMI (1 b = 0 image + 64 directions with b = 1,000 s/*mm*^2^), the first steps in our preprocessing pipeline involve FSL's (Smith et al., 2004) *topup* tool (Andersson et al., 2003) to unwarp deformations caused by magnetic field inhomogeneities, followed by *eddy* for correcting distortions caused by eddy currents. Next, we perform bias field correction using the implementation provided by MRtrix3 (Tournier et al., 2019), based on the method from Raffelt et al. (2017). Finally, we regrid the resulting DWI images to an isotropic voxel resolution of 1.25 mm.

#### 2.2.2 Co-registration

To sample connectome metrics from comparable brain regions across different subjects, the parcellation atlas and the current subject of interest must reside in the same coordinate space. In other words, either the parcellation atlas must be registered to the DWI space of the subject, or the DWI signal must be registered to the template space of the parcellation atlas (in this work, the MNI space). While the latter option has the advantage that all tractograms will be generated in the same space across subjects, we choose the former option since it preserves real-world distances in DWI space. In that sense, we co-register the AAL atlas from MNI to DWI subject space by following the steps illustrated in Figure 1 and outlined below.

1. T1 to MNI space: ANTs registration hierarchy of affine registration (7 degrees of freedom) followed by a nonlinear registration.
2. DWI to T1: Affine registration is sufficient because distortions in the diffusion data were corrected, and both scans were acquired from the same subject in the same session.
3. Then, the respective transformations between spaces were applied backwards to project the AAL atlas from MNI into subject DWI space (i.e., MNI to T1 to DWI) and to project the T1 image into DWI space. This registration also accounts for subject movements between acquisitions of the different modalities.

#### 2.2.3 Response function estimation and spherical deconvolution

To estimate the response functions necessary for spherical deconvolution, we employ the Dhollander algorithm (Dhollander et al., 2019), specifically designed for response function estimation from single-shell diffusion-weighted imaging (DWI) data, including the b0 image. This method facilitates the extraction of tissue-specific response functions for spherical deconvolution. Following the recommendations of MRtrix3, these response functions were averaged using the healthy controls.^1^ Following this, we utilize the Single-Shell 3-Tissue Constrained Spherical Deconvolution (CSD) approach (Dhollander and Connelly, 2016) using the averaged response functions, which enables the differentiation of multiple tissue types within a single-shell DWI dataset, leading to the reconstruction of fiber orientation distributions (FOD). This functionality is provided by a fork of MRtrix3 called MRtrix3Tissue (https://3Tissue.github.io). Tissue-selective FOD images are retrieved for WM, gray matter GM, and cerebrospinal fluid (CSF). The WM FODs represent the underlying WM fiber architecture and are used for subsequent tractography analyses.

#### 2.2.4 Tractography

In fiber orientation distribution (FOD)-guided streamline tractography, pathways are iteratively traced from seed points to terminal regions by following the FOD field. In this work, seed points are identified using a dynamic seeding method, which leverages the SIFT2 (Smith et al., 2015) model to find viable seeding locations. We utilize the iFOD2 algorithm, a probabilistic tractography method. Unlike deterministic approaches, probabilistic tractography randomly samples directions around the peaks of the local FOD signal, allowing it to account for the noise and variability inherent in diffusion-weighted imaging (DWI) data. This approach effectively captures complex fiber configurations, such as crossing fibers, which deterministic methods may oversimplify. Additionally, Anatomically Constrained Tractography (ACT) (Smith et al., 2012) is applied using five-tissue-type segmentation based on the T1-weighted image in DWI subject space, further refining the accuracy of the generated tractograms since only anatomically plausible streamlines are considered.

#### 2.2.5 Tractogram filtering with SIFT2

Tractography generally suffers from low specificity, leading to a significant number of false-positive streamlines that lack a corresponding biological neural bundle counterpart. To address this issue, tractogram filtering methods have been developed to remove or assign lower weights to these faulty streamlines. Various filtering methods are categorized based on their assessment of streamline quality, as explicitly divided by Jörgens et al. (2021). These categories include the explainability of diffusion signals (by inverting the DWI-to-tractogram operation), the use of inclusion and exclusion regions of interest (ROIs), streamline geometry or shape, and streamline similarity and clustering. Among these, methods focusing on the explainability of the diffusion signal are particularly beneficial for structural connectomics because they aim to enhance the consistency of the filtered tractograms with the acquired diffusion data. One widely used method based on this approach is Spherical-deconvolution Informed Filtering of Tractograms (SIFT) (Smith et al., 2013), along with its successor, SIFT2 (Smith et al., 2015), which was employed in this study.

SIFT works by selecting streamlines from the tractogram that best reconstruct the fiber densities across the brain. It iteratively calculates each streamline's contribution to the diffusion signal in every voxel it traverses, removing those that contribute the least to the overall signal. Several termination criteria can be applied to stop this process, such as reaching a user-defined limit for the number of remaining streamlines (Smith et al., 2013). In contrast, the SIFT2 algorithm does not remove streamlines but assigns weights to those with smaller contributions to the diffusion signal (Smith et al., 2015). Thus, SIFT2 weights are used to create weighted averages that are used to construct connectivity matrices. Both methods were developed by the same research group and they recommend using SIFT2 for connectivity analyses. Consequently, we utilize weighted connectivity matrices for further processing and interpret the network connectome as a weighted, undirected graph.

Based on the implications of incorporating SIFT2 into the connectome generation, we evaluate our complete experimental framework by comparing the connectomes with and without the application of SIFT2—referred to as unfiltered and filtered, respectively.

#### 2.2.6 Connectome sampling

From the unfiltered and filtered tractograms and the individual parcellation atlases, the connectivity matrices were computed using the *tck2connectome* script from MRTrix3. This script identifies the endpoints of each streamline and assigns them to the nearest brain regions. The resulting connectivity matrices are square adjacency matrices (90 × 90), where the elements represent the edges between the brain regions in each column and row. Given that structural connectomics deduces connectivity without indicating the nature of the connection, connections between brain regions are undirected.

We evaluate several options to define the contribution of each streamline to the weights of the edges in the connectivity matrix (Calamante, 2017). Specifically, we computed connectivity matrices based on the raw streamline count (SC) as well as using the streamlines to sample different diffusion tensor metrics at the points along the streamlines. For the latter, we used maps of fractional anisotropy (FA), mean diffusivity (MD), radial diffusivity (RD), and axial diffusivity (AD). In this case, every streamline is associated with the mean value of the metric (e.g., FA) along the path of the streamline.

Note that the presence of noise in the data and the application of probabilistic tractography, in accordance with the intrinsic biases of structural connectivity itself, lead to a certain number of false positives in the tractogram. Moreover, since we refrain from thresholding the network graphs, the resulting connectomes are densely connected. However, we expect the effects of these false positives not to be detrimental based on two arguments. First, node pairs that are falsely connected are expected to share a lower absolute number of streamlines and, therefore, are associated with low values in the SC-based connectomes. Since the connectivity measure of track-weighted connectomes (e.g., FA and MD) is largely averaged: along the path of every streamline and across all streamlines connecting pairs of regions, false positives are expected to have less impact on connectivity estimations. Second, in cases where false positives have an effect, we expect filtering to lead to an observable improvement, as it is designed to assign lower values to streamlines that do not contribute to the reconstruction of the initial DWI signal.

### 2.3 Statistical analysis of connectomes

To assess the discriminative power of connectome values (brain network connections), we evaluate threshold-free network-based statistics (TFNBS) as proposed by (Baggio et al. 2018). TFNBS is an advancement over NBS (Zalesky et al., 2010), as it accumulates statistics across a range of thresholding steps for binarizing the t-statistic rather than relying on a predefined threshold level. Additionally, both TFNBS and NBS allow for family-wise error rate correction of p-values, which is less restrictive than conventional multiple comparison corrections applied directly to edge-wise statistics. This approach alters the interpretation by evaluating the significance with respect to the extent (number of connections) and height (current threshold) of connected components across different threshold values, rather than considering edge statistics independently. Moreover, we test both hypotheses for all connectivity metrics as follows: hypothesis t1: PD > HC, hypothesis t2: HC > PD.

### 2.4 Graph theory measures at node and whole-brain levels

To assess the distinguishing properties of graph theory-related metrics in the derived brain networks, we utilize the following graph measures: node strength, betweenness centrality, and clustering coefficient. Node strength in a weighted undirected graph is the sum of edge weights between a node and its neighboring nodes. It is a local metric that is useful for identifying hubs in the network (nodes with high connectivity). The betweenness centrality of a node measures the proportion of all shortest paths in the network that pass through that node. This metric indicates the node's significance for global, network-wide information transfer. The clustering coefficient is a localized metric that quantifies the degree of interconnectedness between a node and its neighbors. Specifically, it measures the proportion of a node's neighbors that are also connected to each other, reflecting the level of interconnectivity in the node's immediate neighborhood. Lastly, we report assortativity, a global metric that measures the correlation between the node strengths of connected nodes, indicating whether nodes with similar strengths tend to connect to each other.

### 2.5 Classification

We used the connectomes to perform binary classification between PD vs. HC. For this, we use support vector machines (SVM) and graph neural networks (GNNs).

#### 2.5.1 Support vector machine with feature reduction

We chose SVM as the first kind of model architecture due to its simplicity, good generalizability, acceptance, and history in the field of machine learning. In our experiments, we applied SVM alone on the upper diagonal entries of the connectomes or after selecting the 64 most relevant features extracted with principal component analysis (PCA). We referred to the latter model as SVM64 in the experiments. We utilized the implementation provided by *scikit-learn* (Pedregosa et al., 2011). We used a kernel-based SVM with a linear kernel trained with 5-fold cross-validation.

#### 2.5.2 Graph neural networks

In contrast to SVM, graph neural networks (GNNs) offer a more complex model architecture with the benefit of directly learning on the underlying structure (i.e., the graph itself). In addition, the deep multi-layer configuration of GNNs enables the learning of more expressive relationships between data entries, although it is also more prone to overfitting than the simple SVM. We used two types of GNN: *graph attention networks* (GATs) and *graph convolutional networks* (GCNs). On the one hand, GATs provide an additional attention mechanism that has recently been shown to aid in learning problems in deep learning, in general, and in GNNs, in particular (Veličković et al., 2017). By widely following the recommendations by Cui et al. (2023), we make the following concrete design choices for the models tested in this study:

##### 2.5.2.1 Message passing function

Based on the high-performance scores on subjects from the same database (PPMI) with a similar methodology, we decided to utilize *node concat* message passing for GCN and *node concat with attention* for GAT (Equations 1 and 2):


$$\begin{array}{c} \psi \left(x_{i},x_{j}\right)=\psi_{\text{MLP}}\left(x_{i}||x_{j}\right), \end{array}$$ (1)

$$\begin{array}{c} \psi \left(x_{i},x_{j}\right)=\psi_{\text{MLP}}\left(x_{i}||\left(x_{j}a_{ij}\right)\right), \end{array}$$ (2)

where, ψ represents the message-passing function for GCN or GAT, *x*_*i*_ is the feature vector at node *i*, ψ_MLP_ denotes a multi-layered perceptron (MLP) and || indicates the vector concatenation operation. For GAT, *a*_*ij*_ represents the attention coefficient between nodes *i* and *j*.

##### 2.5.2.2 Node connection profile

Connectomes as described in Section 2.2.6 are edge-features on the brain network graph. Conventionally, GNNs take node features as inputs for classification. Therefore, a conversion is required between edge and node features here. We again follow the suggestion from Cui et al. (2023) and construct node features based on connection profiles. This means that each node receives an input feature vector consisting of the connectome weights between this node and all other nodes. This methodology has been used in other studies (Ktena et al., 2018).

## 3 Results

### 3.1 Connection level evaluation

We tested two hypotheses independently. The first hypothesis (t1) proposed that connectivity measures (SC, FA, AD, MD, and RD) would be higher within the Parkinson's Disease (PD) cohort than in healthy controls (HC) on specific brain network edges (brain-region pairs). The second hypothesis (t2) implied these measures would be higher in HC than in PD. Different nodes achieved the highest discriminability across the various measures. We interpret the t-statistics and the resulting *p-*values to gauge how well these metrics distinguish between PD and HC.

Similar to previous studies, we report both uncorrected and corrected p-values to provide readers with an idea of how discriminative the two groups are on specific metrics or network edges. Table 2 shows the three most significant region pairs for unfiltered connectomes with SC for hypothesis t1, which are the only three that survived the Benjamini-Yekutieli (BY) correction. These connections did not survive BY correction when SIFT2 was applied. The ten most significant connections for all types of connectomes with and without filtering and the two hypotheses are shown in Appendix Tables A.1–A.20. The ranking order of node region pairs changed when filtered connectomes were compared to those based on unfiltered tractography, although the highest-scoring pairs remained among the top-scoring regardless of filtering. This indicates that SIFT2 retains the strongest connections. Moreover, we did not observe a consistent improvement in discriminability between the groups when using SIFT2. We report effect sizes in Appendix Tables A.1–A.20.

**Table 2:** Unfiltered connectomes, metric: SC, hypothesis t1: PD > HC.

Connection	uncor. P (t1)	FWE P (t1)	t val. (t1)
Amygdala L↔Occipital Mid L	0.0006	0.0424	3.5996
Frontal Inf Oper L↔Occipital Mid L	0.0004	0.0498	3.5033
Occipital Mid L↔Paracentral Lobule L	0.0004	0.0498	3.5310

### 3.2 Node level evaluation

**Figure 2:**
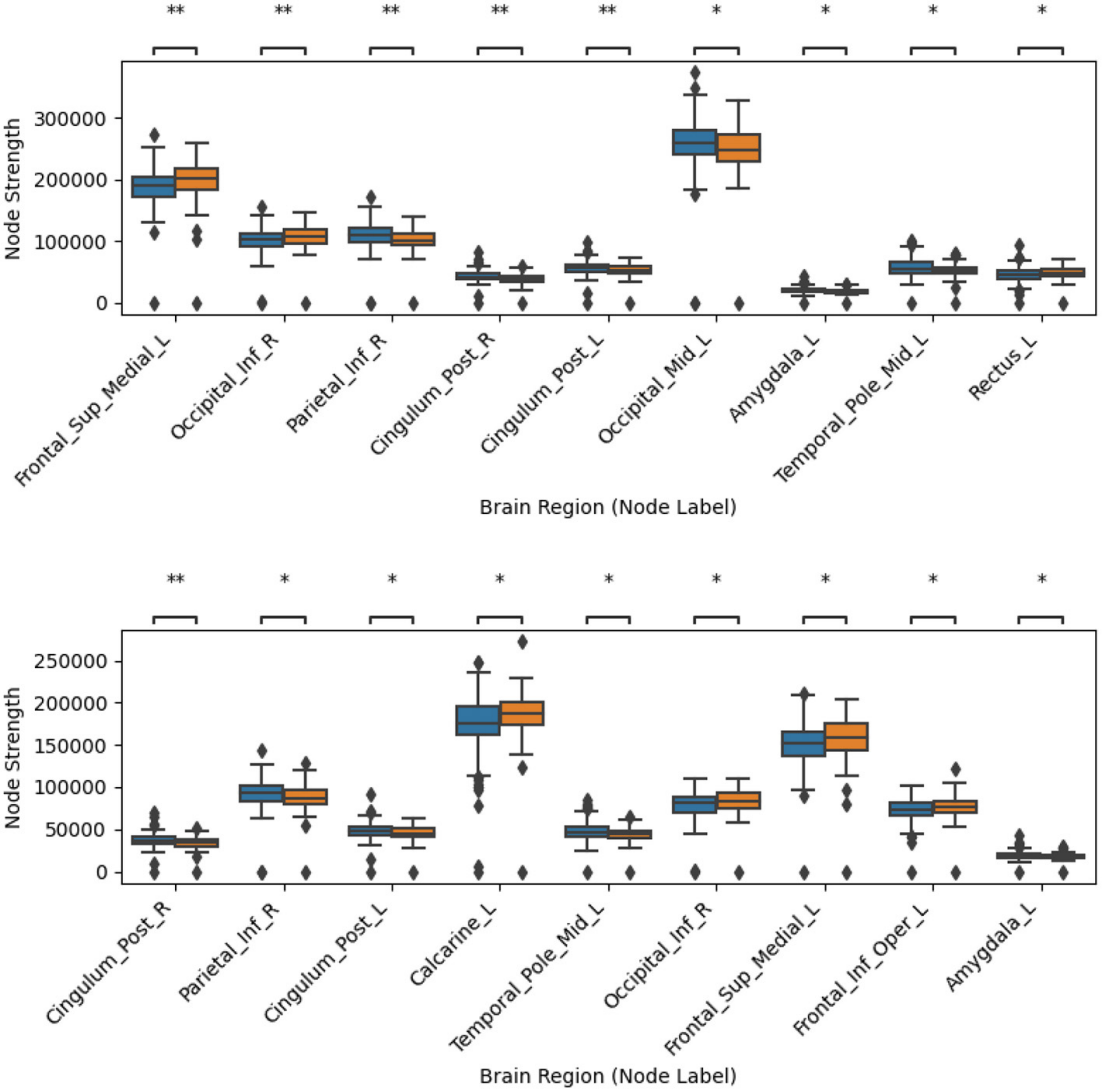
Comparison of node strength error bars for PD vs. HC using the streamline counting (SC) metric. The top panel shows results without streamline filtering, while the bottom panel shows results with streamline filtering using SIFT2. Nodes are ordered from left to right based on their discriminability, with lower p-values on the left. Only node regions significant before family-wise error correction using the Benjamini-Yekutieli (BY) procedure are displayed. PD and HC subjects are depicted in blue and orange, respectively. ^**^ indicates *p* < 0.01 before correction, * indicates *p* < 0.05 before correction. The results after correction for multiple comparisons across all 90 nodes were not significant for any of the nodes.

**Figure 3:**
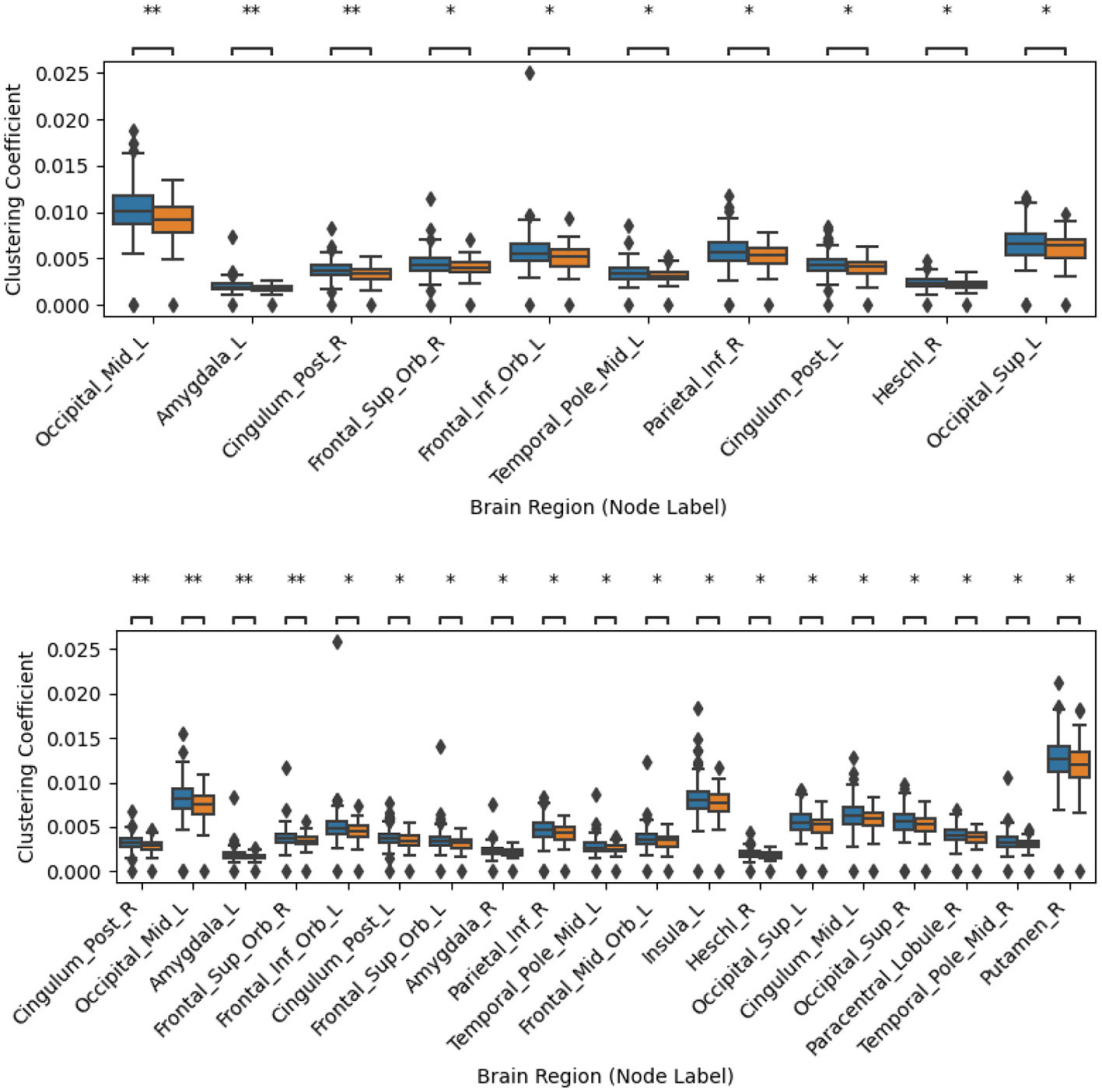
Comparison of clustering coefficient error bars for PD vs. HC using the streamline counting (SC) metric. The top panel shows results without streamline filtering, while the bottom panel shows results with streamline filtering using SIFT2. Nodes are ordered from left to right based on their discriminability, with lower p-values on the left. Only node regions significant before family-wise error correction using the Benjamini-Yekutieli (BY) procedure are displayed. PD and HC subjects are depicted in blue and orange, respectively. ** indicates *p* < 0.01 before correction, * indicates *p* < 0.05 before correction. The results after correction for multiple comparisons across all 90 nodes were not significant for any of the nodes.

**Figure 4:**
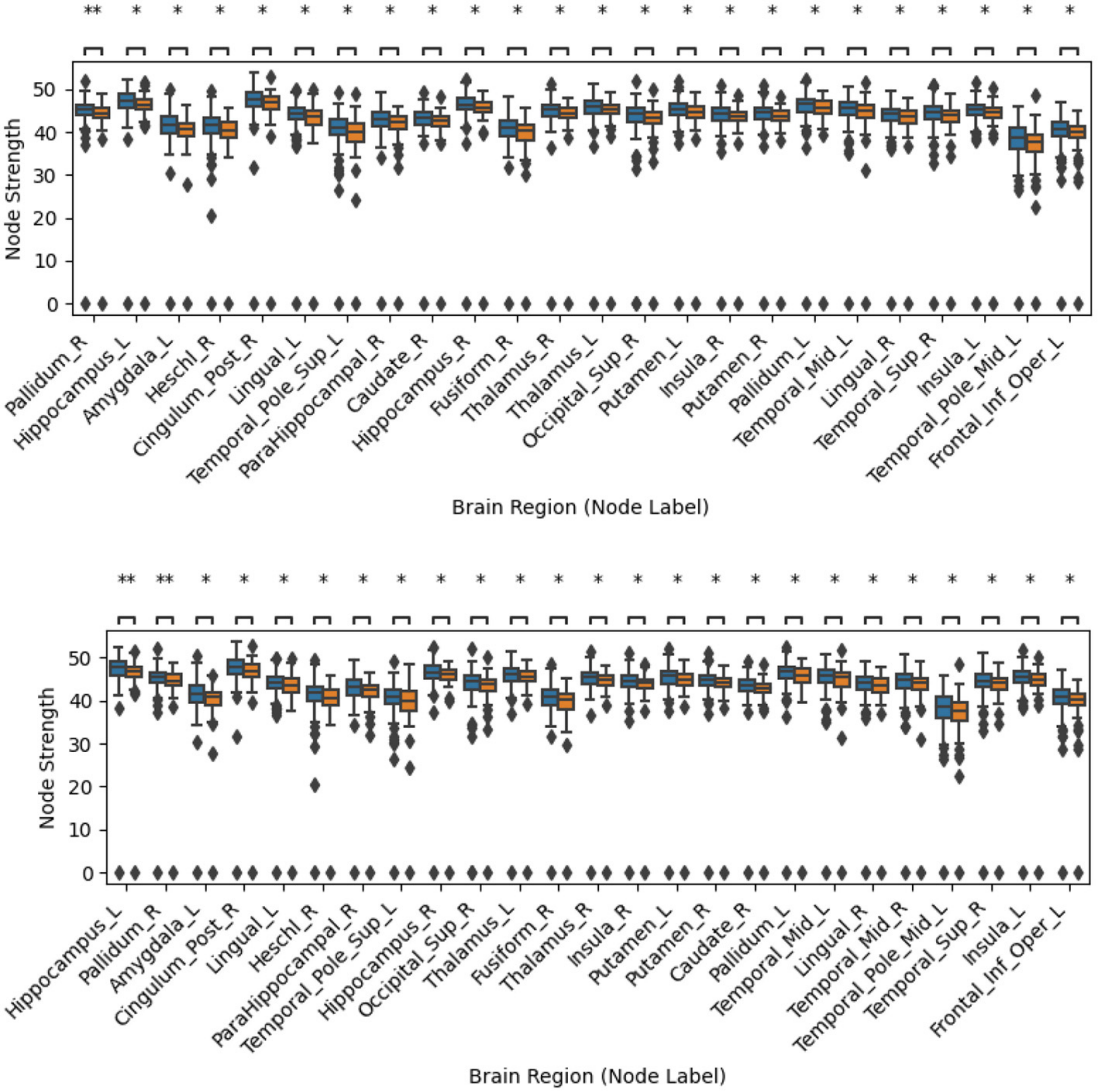
Comparison of node strength error bars for PD vs. HC using the fractional anisotropy (FA) metric. The top panel shows results without streamline filtering, while the bottom panel shows results with streamline filtering using SIFT2. Nodes are ordered from left to right based on their discriminability, with lower p-values on the left. Only node regions significant before family-wise error correction using the Benjamini-Yekutieli (BY) procedure are displayed. PD and HC subjects are depicted in blue and orange, respectively. ^**^ indicates *p* < 0.01 before correction, * indicates *p* < 0.05 before correction. The results after correction for multiple comparisons across all 90 nodes were not significant for any of the nodes.

Appendix Figures A.2, A.3, A.5, A.6, A.8, A.9, A.11, A.12, A.14, A.15 show the most discriminative nodes for the three graph node metrics, both for filtered and unfiltered tractograms for the five different ways of creating tractograms. The indicated significance levels were corrected for multiple comparisons with the BY method. Figures 2–4 display an extract of those figures. As shown, none of the differences remained significant after this correction. Tables 3–5 summarize the main findings from the figures in the Appendix. As shown, the results with and without tractogram filtering are very similar, in most cases, only affecting the level of significance. These tables also show that the tractograms created with the five different metrics yield different results. This result was expected, as tractograms created with different metrics look for different hypotheses. Indeed, while FA, AD, RD, and MD are extracted from DTI, every metric conveys different local information. In turn, SC is usually used as a surrogate for the strength of connections. Thus, they can be seen as complementary to each other.

**Table 3:** Brain regions with statistically significant differences in the node strength before correcting for multiple comparisons.

Node	SC		FA		AD		RD		MD	
	U	F	U	F	U	F	U	F	U	F
Frontal_Sup_Medial_L	H^*^^*^	H^*^	-	-	-	-	-	-	-	-
Occipital_Inf_R	H^*^^*^	H^*^	-	-	-	-	-	-	-	-
Parietal_Inf_R	P^*^^*^	P^*^	-	-	-	-	-	-	-	-
Cingulum_Post_R	P^*^^*^	P^*^^*^	P^*^	P^*^	-	-	-	-	-	-
Cingulum_Post_L	P^*^^*^	P^*^	-	-	-	-	-	-	-	-
Occipital_Mid_L	P^*^	-	-	-	-	-	-	-	-	-
Amygdala_L	P^*^	P^*^	P^*^	P^*^	-	-	-	-	-	-
Temporal_Pole_Mid_L	P^*^	P^*^	P^*^	P^*^	-	-	-	-	-	-
Rectus_L	H^*^	-	-	-	-	-	-	-	-	-
Calcarine_L	-	H^*^	-	-	-	-	-	-	-	-
Frontal_Inf_Oper_L	-	H^*^	P^*^	P^*^	-	-	-	-	-	-
Pallidum_R	-	-	P^*^^*^	P^*^^*^	-	-	H^*^	-	-	-
Hippocampus_L	-	-	P^*^	P^*^^*^	-	-	-	-	-	-
Heschl_R	-	-	P^*^	P^*^	-	-	-	-	-	-
Lingual_L	-	-	P^*^	P^*^	-	-	-	-	-	-
Temporal_Pole_Sup_L	-	-	P^*^	P^*^	-	-	-	-	-	-
ParaHippocampal_R	-	-	P^*^	P^*^	-	-	-	-	-	-
Caudate_R	-	-	P^*^	P^*^	-	-	-	-	-	-
Hippocampus_R	-	-	P^*^	P^*^	-	-	-	-	-	-
Fusiform_R	-	-	P^*^	P^*^	-	-	-	-	-	-
Thalamus_R	-	-	P^*^	P^*^	-	-	-	-	-	-
Thalamus_L	-	-	P^*^	P^*^	-	-	-	-	-	-
Occipital_Sup_R	-	-	P^*^	P^*^	-	-	-	-	-	-
Putamen_L	-	-	P^*^	P^*^	-	-	-	-	-	-
Insula_R	-	-	P^*^	P^*^	-	-	-	-	-	-
Putamen_R	-	-	P^*^	P^*^	-	-	-	-	-	-
Pallidum_L	-	-	P^*^	P^*^	-	-	-	-	-	-
Temporal_Mid_L	-	-	P^*^	P^*^	-	-	-	-	-	-
Lingual_R	-	-	P^*^	P^*^	-	-	-	-	-	-
Temporal_Sup_R	-	-	P^*^	P^*^	-	-	-	-	-	-
Insula_L	-	-	P^*^	P^*^	-	-	-	-	-	-
Temporal_Mid_R	-	-	-	P^*^	-	-	-	-	-	-
Frontal_Sup_Orb_L	-	-	-	-	-	-	H^*^	H^*^	H^*^	H^*^
Angular_R	-	-	-	-	-	-	H^*^	H^*^	-	-

SC, FA, AD, RD, and MD correspond to the different metrics used to create the connectomes. U and F refer to unfiltered and filtered tractograms, respectively. H denotes outcomes where the mean ranking of the node strength from healthy controls is higher than PD subjects, while P denotes the opposite, and "-" is used for no statistical differences. One or two asterisks show uncorrected *p*-values below 0.05 and 0.01, respectively.

**Table 4:** Brain regions with statistically significant differences in the betweenness centrality before correcting for multiple comparisons.

Node	SC		FA		AD		RD		MD	
	U	F	U	F	U	F	U	F	U	F
Occipital_Inf_R	H^**^	-	-	-	-	-	-	-	-	-
Hippocampus_L	H^*^	H^*^	-	-	-	-	-	-	-	-
Occipital_Mid_L	P^*^	-	-	-	-	-	-	-	-	-
Parietal_Inf_R	P^*^	P^*^	-	-	-	-	-	-	-	-
Fusiform_L	H^*^	-	-	-	-	P^*^	-	-	-	-
SupraMarginal_L	P^*^	-	-	-	-	-	-	-	-	-
Temporal_Pole_Sup_L	-	-	H^**^	-	-	-	-	-	-	-
Occipital_Inf_L	-	-	P^*^	P^*^	-	-	-	-	-	-
Frontal_Mid_Orb_L	-	-	H^*^	-	-	-	-	-	-	-
Putamen_L	-	-	-	H^*^	-	-	-	-	-	-
Precentral_L	-	-	-	-	H^**^	H^**^	-	-	-	-
Occipital_Mid_R	-	-	-	-	H^**^	H^*^	-	-	-	-
Occipital_Sup_L	-	-	-	-	H^**^	H^*^	-	-	-	-
Cingulum_Mid_L	-	-	-	-	H^*^	-	-	-	-	H*
Thalamus_R	-	-	-	-	-	H^**^	-	-	-	H*
Temporal_Sup_R	-	-	-	-	-	H^**^	-	-	-	-
Frontal_Inf_Oper_R	-	-	-	-	-	H^*^	-	-	-	-
Postcentral_R	-	-	-	-	-	H^*^	-	P^*^	-	-
Putamen_R	-	-	-	-	-	-	H^*^	-	-	-
Frontal_Mid_R	-	-	-	-	-	-	-	P^*^	-	-
Frontal_Inf_Tri_R	-	-	-	-	-	-	-	-	P^*^	P^*^

SC, FA, AD, R,D and MD correspond to the different metrics used to create the connectomes. U and F refer to unfiltered and filtered tractograms, respectively. H denotes outcomes where the mean ranking of the betweenness centrality from healthy controls is higher than that of PD subjects, while P denotes the opposite, and "-" is used for no statistical differences. One or two asterisks show uncorrected p-values below 0.05 and 0.01, respectively.

**Table 5:** Brain regions with statistically significant differences in the clustering coefficient before correcting for multiple comparisons.

Node	SC		FA		AD		RD		MD	
	U	F	U	F	U	F	U	F	U	F
Occipital_Mid_L	P^**^	P^**^	-	-	-	-	-	-	-	-
Amygdala_L	P^**^	P^**^	-	-	-	-	-	-	-	-
Cingulum_Post_R	P^**^	P^**^	-	-	-	-	-	-	-	-
Frontal_Sup_Orb_R	P^*^	P^**^	-	-	-	-	-	-	-	-
Frontal_Inf_Orb_L	P^*^	P^*^	-	-	-	-	-	-	-	-
Temporal_Pole_Mid_L	P^*^	P^*^	-	-	-	-	-	-	-	-
Parietal_Inf_R	P^*^	P^*^	-	-	-	-	-	-	-	-
Cingulum_Post_L	P^*^	P^*^	-	-	-	-	-	-	-	-
Heschl_R	P^*^	P^*^	-	-	-	-	-	-	-	-
Occipital_Sup_L	P^*^	P^*^	-	-	-	-	-	-	-	-
Frontal_Sup_Orb_L	-	P^*^	-	-	-	-	-	-	-	-
Amygdala_R	-	P^*^	-	-	-	-	-	-	-	-
Frontal_Mid_Orb_L	-	P^*^	-	-	-	-	-	-	-	-
Insula_L	-	P^*^	-	-	-	-	-	-	-	-
Cingulum_Mid_L	-	P^*^	-	-	-	-	-	-	-	-
Occipital_Sup_R	-	P^*^	-	-	-	-	-	-	-	-
Paracentral_Lobule_R	-	P^*^	-	-	-	-	-	-	-	-
Temporal_Pole_Mid_R	-	P^*^	-	-	-	-	-	-	-	-
Putamen_R	-	P^*^	-	-	-	-	-	-	-	-

SC, FA, AD, RD, and MD correspond to the different metrics used to create the connectomes. U and F refer to unfiltered and filtered tractograms, respectively. H denotes outcomes where the mean ranking of the clustering coefficient from healthy controls is higher than that of PD subjects, while P denotes the opposite, and "-" is used for no statistical differences. One or two asterisks show uncorrected p-values below 0.05 and 0.01, respectively.

### 3.3 Whole-brain evaluation

Connectome-wise metrics from averaging over all nodes did not result in statistically significant differences (see Appendix Figures A.1, A.4, A.7, A.10, A.13). Appendix Table A.21 shows effect sizes for these metrics. As shown, the effect sizes are small to negligible (|*d*| < 0.3), and the 95% confidence intervals consistently include zero, indicating no meaningful group separation. The change in effect size due to filtering (Δ*d*) is uniformly near zero (mean |Δ*d*| = 0.027), demonstrating that SIFT2 filtering does not alter the (already negligible) group differences.

### 3.4 Classification performance evaluation

Given the class imbalance between the PD and control groups, the area under the receiver operating characteristic curve (AUC) was selected for performance assessment in these experiments. Table 6 presents the AUC values for all tested classification methods, which uniformly demonstrated poor classification performance. The AUC values were close to 0.5, indicating that the classifiers performed at a level similar to random chance.

**Table 6:** Classification metrics from various connectome methods and classification models.

Filtering	Metric	Classifier	Accuracy	Precision	Recall	AUC	F1
None	SC	GAT	0.62 ± 0.07	0.35 ± 0.09	**0.36** **±0.10**	0.54 ± 0.06	**0.35** **±0.08**
		GCN	0.66 ± 0.06	0.37 ± 0.13	0.30 ± 0.15	**0.55** **±0.09**	0.33 ± 0.14
		SVM	**0.70** **±0.04**	**0.46** **±0.21**	0.15 ± 0.05	0.54 ± 0.04	0.22 ± 0.08
		SVM64	0.64 ± 0.04	0.34 ± 0.11	0.31 ± 0.16	0.54 ± 0.07	0.32 ± 0.13
	FA	GAT	0.57 ± 0.07	0.20 ± 0.14	0.22 ± 0.19	0.47 ± 0.07	0.20 ± 0.16
		GCN	0.62 ± 0.03	0.31 ± 0.04	0.25 ± 0.05	0.51 ± 0.03	0.28 ± 0.04
		SVM	0.69 ± 0.03	0.35 ± 0.42	0.05 ± 0.04	0.50 ± 0.03	0.08 ± 0.07
		SVM64	0.63 ± 0.05	0.27 ± 0.16	0.18 ± 0.14	0.50 ± 0.06	0.21 ± 0.13
	AD	GAT	0.58 ± 0.03	0.21 ± 0.14	0.24 ± 0.19	0.48 ± 0.05	0.22 ± 0.16
		GCN	0.61 ± 0.04	0.30 ± 0.05	0.25 ± 0.05	0.50 ± 0.03	0.27 ± 0.04
		SVM	0.68 ± 0.04	0.13 ± 0.30	0.03 ± 0.07	0.49 ± 0.05	0.05 ± 0.11
		SVM64	0.62 ± 0.02	0.21 ± 0.13	0.15 ± 0.11	0.48 ± 0.03	0.17 ± 0.11
	RD	GAT	0.56 ± 0.05	0.23 ± 0.11	0.24 ± 0.13	0.47 ± 0.07	0.23 ± 0.12
		GCN	0.60 ± 0.09	0.23 ± 0.14	0.14 ± 0.07	0.46 ± 0.08	0.17 ± 0.09
		SVM	0.69 ± 0.03	0.22 ± 0.22	0.05 ± 0.04	0.50 ± 0.02	0.07 ± 0.07
		SVM64	0.62 ± 0.06	0.27 ± 0.14	0.17 ± 0.07	0.49 ± 0.06	0.20 ± 0.09
	MD	GAT	0.58 ± 0.05	0.25 ± 0.10	0.24 ± 0.11	0.48 ± 0.05	0.24 ± 0.09
		GCN	0.61 ± 0.05	0.28 ± 0.07	0.21 ± 0.06	0.49 ± 0.03	0.23 ± 0.04
		SVM	0.68 ± 0.05	0.07 ± 0.15	0.02 ± 0.03	0.48 ± 0.03	0.03 ± 0.06
		SVM64	0.63 ± 0.04	0.24 ± 0.10	0.14 ± 0.07	0.48 ± 0.04	0.17 ± 0.08
SIFT2	SC	GAT	0.59 ± 0.06	0.29 ± 0.11	**0.33** **±0.17**	0.51 ± 0.07	0.30 ± 0.13
		GCN	0.68 ± 0.05	0.46 ± 0.12	0.31 ± 0.10	**0.57** **±0.02**	**0.35** **±0.07**
		SVM	0.69 ± 0.05	0.36 ± 0.25	0.12 ± 0.10	0.52 ± 0.07	0.18 ± 0.15
		SVM64	0.61 ± 0.05	0.29 ± 0.10	0.22 ± 0.05	0.49 ± 0.05	0.25 ± 0.07
	FA	GAT	0.64 ± 0.09	0.36 ± 0.27	0.18 ± 0.08	0.51 ± 0.09	0.23 ± 0.13
		GCN	0.58 ± 0.05	0.18 ± 0.06	0.14 ± 0.07	0.45 ± 0.04	0.15 ± 0.06
		SVM	**0.70** **±0.01**	**0.50** **±0.29**	0.07 ± 0.00	0.52 ± 0.01	0.13 ± 0.00
		SVM64	0.63 ± 0.07	0.28 ± 0.19	0.18 ± 0.14	0.50 ± 0.06	0.21 ± 0.13
	AD	GAT	0.57 ± 0.06	0.23 ± 0.14	0.22 ± 0.15	0.46 ± 0.08	0.22 ± 0.14
		GCN	0.64 ± 0.02	0.32 ± 0.06	0.24 ± 0.06	0.52 ± 0.03	0.27 ± 0.06
		SVM	0.69 ± 0.03	0.24 ± 0.25	0.08 ± 0.09	0.51 ± 0.04	0.11 ± 0.13
		SVM64	0.63 ± 0.03	0.20 ± 0.15	0.13 ± 0.12	0.48 ± 0.05	0.16 ± 0.13
	RD	GAT	0.59 ± 0.02	0.20 ± 0.13	0.18 ± 0.15	0.47 ± 0.06	0.19 ± 0.14
		GCN	0.60 ± 0.08	0.26 ± 0.14	0.16 ± 0.06	0.47 ± 0.05	0.19 ± 0.06
		SVM	0.67 ± 0.04	0.10 ± 0.15	0.03 ± 0.04	0.48 ± 0.02	0.04 ± 0.06
		SVM64	0.63 ± 0.05	0.28 ± 0.13	0.17 ± 0.07	0.49 ± 0.05	0.20 ± 0.08
	MD	GAT	0.57 ± 0.06	0.23 ± 0.16	0.24 ± 0.16	0.47 ± 0.08	0.23 ± 0.16
		GCN	0.62 ± 0.07	0.31 ± 0.12	0.22 ± 0.05	0.50 ± 0.06	0.25 ± 0.06
		SVM	0.69 ± 0.03	0.13 ± 0.18	0.03 ± 0.04	0.49 ± 0.02	0.05 ± 0.07
		SVM64	0.62 ± 0.04	0.25 ± 0.09	0.15 ± 0.06	0.48 ± 0.04	0.19 ± 0.07

All metrics are displayed as the mean over 5 stratified folds ± standard deviation. The best-performing models are highlighted independently for filtered and non-filtered tractograms.

Among the connectivity measures, classification using streamline counting achieved marginally better results, with AUC values approximately 5% higher than the other methods. However, this improvement was minimal and did not result in a classification performance that could be considered effective or reliable. Overall, the results indicate that none of the tested classification methods were able to distinguish effectively between the PD and HC groups in this context.

### 3.5 Filtering with SIFT2 in patients and controls

**Figure 5:**
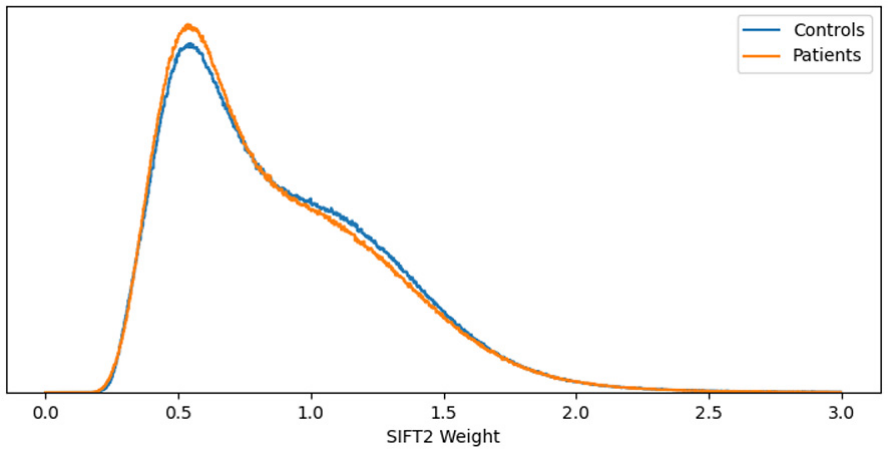
Histogram of SIFT2 weights for 20 randomly selected subjects (10 patients and 10 controls).

In order to assess whether filtering affects more patients or controls, we estimated the histogram of SIFT2 weights from 20 randomly selected subjects. As shown in Figure 5, the histogram for patients and controls is very similar. Thus, filtering appears not to be biased by disease status.

## 4 Discussion

### 4.1 Connection level differences

We evaluated the ability of structural connectivity, based on probabilistic tractography, to discriminate between group differences in Parkinson's patients and healthy controls. Under the various configurations we ran, we found statistical differences that survived BY corrections in three connections connecting the left middle occipital lobe, with patients showing higher values, in connectomes created using streamline counting. However, these connections did not survive BY on filtered tractograms (see Appendix Tables A.1, A.2). One interpretation of these results is that the differences found in the unfiltered tractograms can be caused by faulty streamlines that could affect the connectomes more in PD subjects than in healthy controls. However, since the histogram of SIFT2 values is very similar for patients and controls (see Figure 5), we do not think this is the case in PPMI. Another possibility is that SIFT2 is too strict so subtle differences are removed. (Sarwar et al. 2023) tested different tractogram filtering methods on realistic human-like simulated phantoms and found that filtering improves the quality of tractograms and connectivity analysis. We acknowledge that the results from that study cannot be directly extrapolated to in vivo scenarios. Thus, there is a need to perform validation studies on real and disease-specific data, e.g., using post-mortem data.

### 4.2 Node and whole-brain differences

The most prominent finding is that none of the graph-theoretical metrics we analyzed for individual node regions was strong enough to remain significant after correcting for multiple comparisons. Similarly, global graph metrics, when averaged or accumulated over the entire graph, did not show a significant difference in the distribution of any of the evaluated graph-theoretical measures. Nevertheless, when considering the uncorrected p-values as an indication of discriminability between the groups for each region, the largest group differences were observed in node strength and clustering coefficient from streamline-based connectivity matrices, as well as node strength from fractional anisotropy (FA)-weighted connectomes. Since node strength results from a weighted sum of its connections to neighboring nodes, differences between groups in these regions may reflect a disease-related reorganization. Interestingly, the node strength based on FA was higher in all discriminative node regions for PD compared to HC. While this effect could potentially be explained by compensatory mechanisms, the weak significance observed suggests that further investigation is necessary.

While no statistical differences survived the correction for multiple comparisons, the uncorrected results can be used to assess the effect of tractogram filtering in downstream analyses. We recognize three possible effects: null effect (i.e., the same differences are identified in both filtered and unfiltered connectomes), removal, or emergence of statistical differences. In the case of null effects, one can argue that performing filtering is an unnecessary and expensive step that can be excluded from processing pipelines. As shown in Tables 3–5, this is the most common case, as the analyses with filtered and unfiltered connectomes tend to agree in most of the nodes.

The case of the removal of statistical differences after filtering is more relevant since, in that case, the differences obtained with unfiltered connectomes can be considered artifactual and not related to the disease or caused by removal of true positives by SIFT2. For node strength, this is the case of the Occipital_Mid_L, Rectus_L with SC, and the Pallidum_R with RD, as shown in Table 3. For betweenness centrality (see Table 4), this is the case of the Occipital_Inf_R, Occipital_Mid_L, Fusiform_L, and the SupraMarginal_L with SC, Temporal_Pole_Sup_L, and the Frontal_Mid_Orb_L with FA, and the Putamen_R with RD.

The case of the emergence of new statistical differences after filtering is also relevant. Since SIFT2 has a similar effect on patients and controls (see Figure 5), this can be caused by a large number of faulty streamlines in both groups that can mask relevant differences. For node strength, this is the case of the Calcarine_L and the Frontal_Inf_Oper_L with SC, and the Temporal_Mid_R with FA, as shown in Table 3. For betweenness centrality (see Table 4), this is the case of the Putamen_L with FA, the Fusiform_L, Thalamus_R, Temporal_Sup_R, and the Frontal_Inf_Oper_R with AD, the Postcentral_R and the Frontal_Mid_R for RD, and the Cingulum_Mid_L and the Thalamus_R for the MD. For the clustering coefficient (see Table 5), this is the case of the Frontal_Sup_Orb_L, Amygdala_R Frontal_Mid_Orb_L, Insula_L, Cingulum_Mid_L, Occipital_Sup_R, Paracentral_Lobule_R, Temporal_Pole_Mid_R, and the Putamen_R for SC.

### 4.3 Classification

Classification of patients and controls using structural connectomics can be used not only for clinical purposes, such as diagnosis or disease staging, but also to assess the quality of the connectomes. These applications require models that can predict the groups as accurately as possible.

As shown in Table 6, all combinations of machine learning methods and types of connectomes produced unsatisfactory classification results. Feature reduction with PCA did not result in better results and those are not expected to improve with better handling of the feature reduction process (e.g., Hamdan et al., 2024).

We identified several potential causes for this. First, Parkinson's is a highly heterogeneous disease (Wüllner et al., 2023; Albrecht et al., 2022; Greenland et al., 2019). This can make classification based on structural connectivity more challenging, as different subtypes can affect different brain connections in opposite ways. For comparison, a similar pipeline was successfully applied in Köpff (2020) to classify subjects with mild cognitive impairment (MCI) and healthy controls from structural connectomics, with higher classification accuracy in that context. More recently, Anijärv et al. (2025) employed a similar pipeline to assess correlations between connectivity and asymmetries in tau-PET in Alzheimer's disease. This has also been shown using other types of data for Alzheimer's disease (Miltiadous et al., 2023; Shamrat et al., 2023). Moreover, this has also been reported for other disorders, e.g., mild traumatic brain injury (Huang et al., 2024; Xiao et al., 2015) and different psychiatric disorders (Repple et al., 2023). We acknowledge that ather factors that can influence the classifications are the type of tractography (e.g., Cui et al., 2023 used deterministic), the cohort (e.g., Yang et al., 2021 used a single center in-house dataset), and postprocessing (e.g., thresholding or feature extraction).

We also assessed whether selecting patients with an additional co-morbidity would ease the classification task. For this, we selected a different cohort of patients with both PD and MCI for the classification. In that case, we obtained very similar results to those in Table 6. Our hypothesis is then that the heterogeneity of PD is predominant, making the classification task difficult. It is well-known that filtering methods based on explainability of diffusion signals (like SIFT2) remove redundant streamlines, even if they are plausible (Persson and Moreno, 2024; Hain et al., 2023; Persson et al., 2025). Thus, we cannot rule out the possibility of SIFT2 weighing down too many (redundant) true positives, which can lead to a poor classification performance. However, as mentioned, Sarwar et al. (2023) found that filtering is beneficial for connectivity analysis, at least in realistic phantoms.

Another source of error is the quality of the images. For accurate tractography, multi-shell acquisitions with high spatial and orientation resolution are recommended. A good example of high-quality data is the images acquired by the Human Connectome Project, which contains 3 shells with b-values of 1,000, 2,000, and 3,000 *s*/*mm*^2^ and 90 directions per shell, with a spatial resolution of 1.25mm isotropic. In turn, compared to HCP, the PPMI data is more closely related to clinical acquisitions with a single shell, characterized by a relatively low b-value (1,000), fewer directions (64), and lower resolution (2mm isotropic). That makes it more difficult for tractography to model the fiber bundle configurations in the brain.

It is important to remark that our pipeline follows the recommendations from the field, with parameter configurations selected based on commonly used value ranges and empirical testing. However, other studies in the literature have reported group differences in PPMI data using similar data and methodologies (Yang et al., 2021; Cui et al., 2023; Bergamino et al., 2023). Regarding structural connectivity graph-theory metrics, Zuo et al. (2023) performed a meta-analysis for PD. Using 13 studies, the meta-analysis found group differences in clustering coefficient, characteristic path length, and global efficiency. The heterogeneity (reported as *I*^2^ with the Q-test) was high, moderate, and low, respectively, for these three metrics. Two studies from the meta-analysis are closer to ours. On the one hand, Abbasi et al. (2018) was the only one using PPMI data and reported differences across the three graph-theory metrics. Unlike our study, it used deterministic tractography. In turn, Zarkali et al. (2020) used a pipeline very similar to ours on a private dataset and did not report any statistical differences, consistent with our results. Deterministic tractography was the dominant method in the meta-analysis study (11 vs. 3). Differences among studies can appear due to slight variations in tractography parameters. Therefore, it is possible that the variability in findings across studies highlights the sensitivity of this method to exact model configurations and fine-tuning. Furthermore, cohort selection, study sites, and selection criteria seem to influence outcomes, thereby hindering reproducibility across studies.

### 4.4 Summary of findings

Returning to the four questions posed in the introduction, based on the observations of this study, we conclude the following:

1. **No strong connection-wise significant differences identified:** We identified statistically significant differences between the groups, but these differences disappeared on connectomes from filtered tractograms, which means that they were spurious or tractogram filtering is too strong, so the differences are removed. This outcome suggests that under the specific conditions of our study, structural connectivity, as measured by probabilistic tractography, may not be sufficient to reveal group differences in Parkinson's disease (PD).
2. **No strong node-wise or connectome-wise significant differences identified:** The tested graph theory measures showed changes in specific brain nodes between the groups. These regions with differences were different depending on how the connectome was constructed. That means that the metrics used to create the connectomes convey different information. Thus, combining a multilayer approach in which different connectomes are considered simultaneously could be beneficial for connectomics analyses, just as it is used for combining structural and functional connectivity (e.g., Jauny et al., 2024). In our specific case, none of the statistical differences survived the correction for multiple comparisons. That hints that considering a larger cohort might be necessary to increase the statistical power for PD. We did not find statistically significant differences in graph-theory metrics for the whole brain. Indeed, the possible differences at the node level were averaged out at a larger scale.
3. **Classification model performance:** All of our classification models failed to discriminate between the two classes in the validation sets during the 5-fold cross-validation. This is particularly evident in the AUC measure, where values were close to 0.5 across all classification tasks. We assume this reflects the lack of a discriminative signal, as described above. However, we cannot rule out the possibility that SIFT2 filtering is so strong that it also removes subtle differences between groups. Also, the type of tractography, cohort, and postprocessing can influence the performance of the classifiers.
4. **Tractogram filtering can impact connectomics analyses:** While we did not find statistical differences that survived FWE or correction for multiple comparisons, we found differences between filtered and unfiltered connectomes on graph theory metrics at the node level before correction of p-values. In general, the findings with and without filtering were very similar. However, we found a few disagreements between the findings from filtered and unfiltered connectomes. While studies with phantoms have shown that tractogram filtering increases the reliability of connectivity analyses, more research is needed to assess if those findings can be extrapolated to in vivo imaging.

### 4.5 Limitations

- **Sample size and statistical power:** One potential limitation is the sample size, which may have been insufficient to detect subtle differences. Larger studies might reveal small but meaningful differences that were not detectable in our cohort.
- **Methodological sensitivity:** The study's reliance on a specific configuration of probabilistic tractography and structural connectivity analysis may have limited our ability to detect differences. It would be interesting to exhaustively test the effect of different parameters from the tractography on the results. A limitation of this approach is that these types of analyses are computationally demanding. For example, we used a supercomputer to accelerate data processing, but it still took around three weeks to complete. The same experiments would take approximately 120 days if they were run on a single, powerful workstation.
- **Tractogram filtering methods**: we only tested SIFT2 in this study. A future line of research is to assess the impact of different methods [or their combination, as done in Jörgens et al. (2023)], including randomized approaches (e.g., Hain et al., 2023; Persson et al., 2025) in the analysis of structural connectomes. Also, it would be relevant to assess whether redundancy in tractograms (Persson and Moreno, 2024) influences downstream analyses.

## 5 Conclusion

Based on our observations and the heterogeneous reports from other publications on structural connectivity in Parkinson's disease (see e.g., Samantaray et al., 2022; Sarasso et al., 2021; Zuo et al., 2023), we conclude that structural connectivity analyses for PD are highly sensitive to specific pipeline configurations and fine-tuning. Additionally, the influence of cohort selection and study site differences complicates the reproducibility of results across studies.

## Data Availability

Publicly available datasets were analyzed in this study. This data can be found here: http://www.ppmi-info.org/.

## Footnotes

1
https://mrtrix.readthedocs.io/en/latest/fixel_based_analysis/st_fibre_density_cross-section.html#computing-a-group-average-response-function
