# Supplementary material for: Impact of tractogram filtering and graph creation for structural connectomics in subjects with Parkinson's disease
Source: Front Hum Neurosci. 2026 Apr 23;20:1769103. doi: 10.3389/fnhum.2026.1769103 (PMC13149458; doi:10.3389/fnhum.2026.1769103)
Supplement: Supplementary file 1 [file Data_Sheet_1.pdf]

Appendix

A Comparison of Parkinson's disease patients vs. healthy controls

A.1 Differences in connectomes estimated from streamline counting

Table A.1: 10 most significant connections on unfiltered connectomes, metric: SC, hypothesis t1: PD>HC

Connection	abs. effect (t1)	enh. effect (t1)	uncor. P (t1)	FWE P (t1)	t val. (t1)
Amygdala L↔Occipital Mid L	127.9698	1549.6730	0.0006	**0.0424**	3.5996
Frontal Inf Oper L↔Occipital Mid L	92.3575	1506.7980	0.0004	**0.0498**	3.5033
Occipital Mid L↔Paracentral Lobule L	193.1339	1506.7980	0.0004	**0.0498**	3.5310
Cingulum Post R↔Amygdala L	3.9257	1445.8042	0.0004	0.0594	3.4143
Precentral L↔Occipital Mid L	685.9241	1383.2343	0.0004	0.0708	3.2440
Rolandic Oper L↔Occipital Mid L	118.2167	1383.2343	0.0002	0.0708	3.3372
Amygdala L↔Occipital Sup L	42.3613	1383.2343	0.0006	0.0708	3.2745
Occipital Mid L↔Postcentral L	922.9686	1383.2343	0.0006	0.0708	3.2537
Occipital Mid L↔SupraMarginal L	487.7579	1383.2343	0.0002	0.0708	3.3218
Cingulum Post R↔Thalamus L	37.4665	1304.3216	0.0004	0.0856	3.2106

Table A.2: 10 most significant connections on filtered connectomes, metric: SC, hypothesis t1: PD>HC

Connection	abs. effect (t1)	enh. effect (t1)	uncor. P (t1)	FWE P (t1)	t val. (t1)
Occipital Mid L↔Paracentral Lobule L	115.1992	1316.4566	0.0004	0.0752	3.4609
Precentral L↔Occipital Mid L	402.7696	1241.2156	0.0008	0.0888	3.2445
Frontal Inf Oper L↔Occipital Mid L	49.6552	1197.9780	0.0012	0.0982	3.2150
Rolandic Oper L↔Occipital Mid L	65.7993	1197.9780	0.0004	0.0982	3.1958
Cingulum Post R↔Amygdala L	3.7841	1162.5125	0.0016	0.1096	3.1438
Occipital Mid L↔Postcentral L	520.3096	1146.1088	0.0010	0.1144	3.0955
Occipital Mid L↔SupraMarginal L	315.5940	1146.1088	0.0004	0.1144	3.0709
Frontal Mid Orb L↔Cingulum Post R	0.5827	1132.7215	0.0004	0.1172	3.0799
Amygdala L↔Occipital Sup L	24.6625	1132.7215	0.0006	0.1172	3.1265
Frontal Mid Orb L↔Supp Motor Area L	25.8672	1090.8217	0.0008	0.1318	2.9317

Table A.3: 10 most significant connections on unfiltered connectomes, metric: SC, hypothesis t2: PD<HC

Connection	abs. effect (t2)	enh. effect (t2)	uncor. P (t2)	FWE P (t2)	t val. (t2)
Calcarine L↔Fusiform R	599.1935	960.0970	0.0018	0.4704	3.4087
Calcarine L↔Occipital Inf R	340.0906	924.1600	0.0014	0.5014	3.2647
Frontal Sup Medial L↔Occipital Inf R	0.3695	841.6129	0.0054	0.5728	3.0907
Calcarine L↔Calcarine R	1095.3868	841.6129	0.0026	0.5728	3.1020
Frontal Sup Medial L↔Cingulum Ant L	2754.2754	794.6032	0.0018	0.6164	2.9697
Frontal Sup Medial L↔Hippocampus R	6.9584	748.1750	0.0082	0.6640	2.8479
Frontal Med Orb L↔Rectus L	717.1023	703.2245	0.0038	0.7042	2.7811
Frontal Med Orb L↔Lingual R	0.4724	703.2245	0.0054	0.7042	2.7852
Rectus L↔Hippocampus R	86.5117	703.2245	0.0052	0.7042	2.8024
Calcarine L↔Occipital Inf L	1109.1489	703.2245	0.0044	0.7042	2.8271

Table A.4: 10 most significant connections on filtered connectomes, metric: SC, hypothesis t2: PD<HC

Connection	abs. effect (t2)	enh. effect (t2)	uncor. P (t2)	FWE P (t2)	t val. (t2)
Calcarine L↔Occipital Inf L	1143.0549	928.8049	0.0026	0.4976	3.3524
Calcarine L↔Calcarine R	1111.4086	830.3089	0.0026	0.5820	3.1340
Frontal Sup Medial L↔Occipital Inf R	0.1940	794.6822	0.0044	0.6112	2.9424
Occipital Inf R↔Parietal Sup L	22.0054	794.6822	0.0080	0.6112	2.9385
ParaHippocampal R↔Occipital Inf R	127.9149	762.5007	0.0028	0.6440	2.8916
Calcarine L↔Occipital Inf R	267.4960	762.5007	0.0056	0.6440	2.9065
Calcarine L↔Fusiform R	459.8284	762.5007	0.0038	0.6440	2.8818
Lingual L↔Occipital Inf L	1334.7264	762.5007	0.0030	0.6440	2.8359
Paracentral Lobule L↔Temporal Pole Mid R	0.9529	742.4725	0.0070	0.6616	3.2884
Frontal Sup L↔Frontal Inf Oper L	494.0374	734.0202	0.0042	0.6686	2.8299

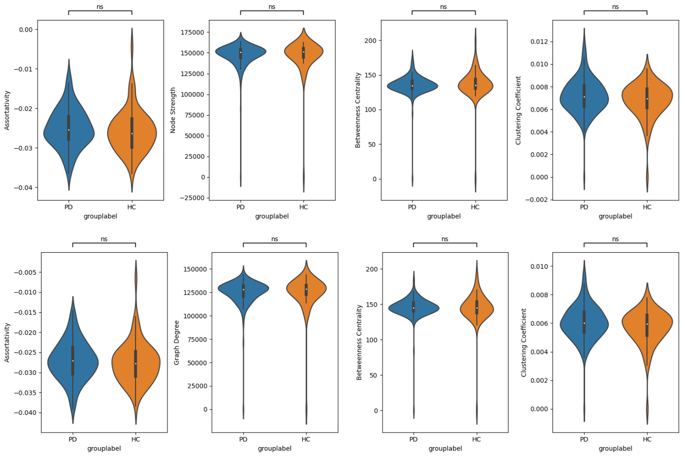

Figure A.1: Distribution of graph theory graph metrics of patients (PD) and healthy controls (HC) on unfiltered connectomes (top) and filtered connectomes (bottom).

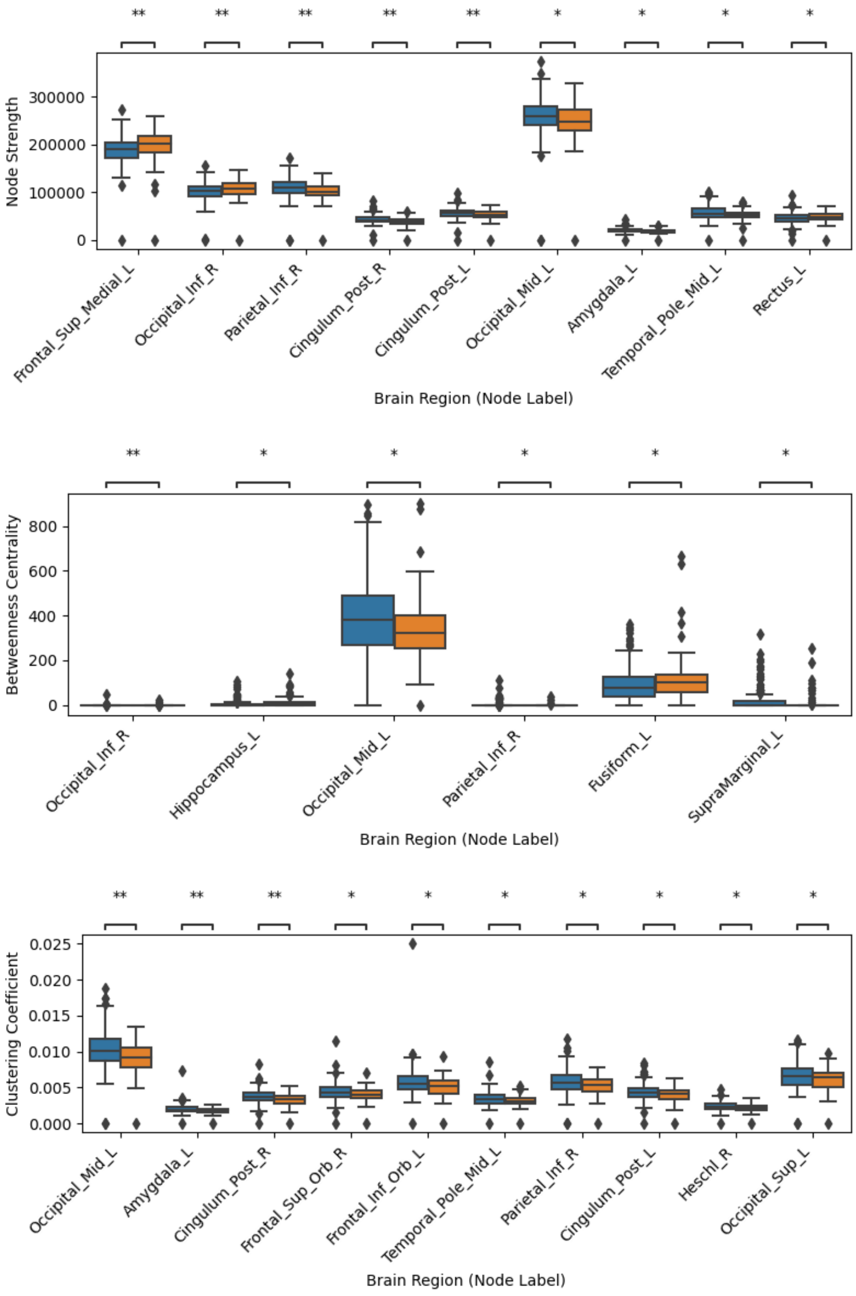

Figure A.2: Distribution of graph theory node metrics of patients (PD) in blue and healthy controls (HC) in orange for the nodes with uncorrected statistical differences for unfiltered connectomes. None of the differences survived correction for multiple comparisons.

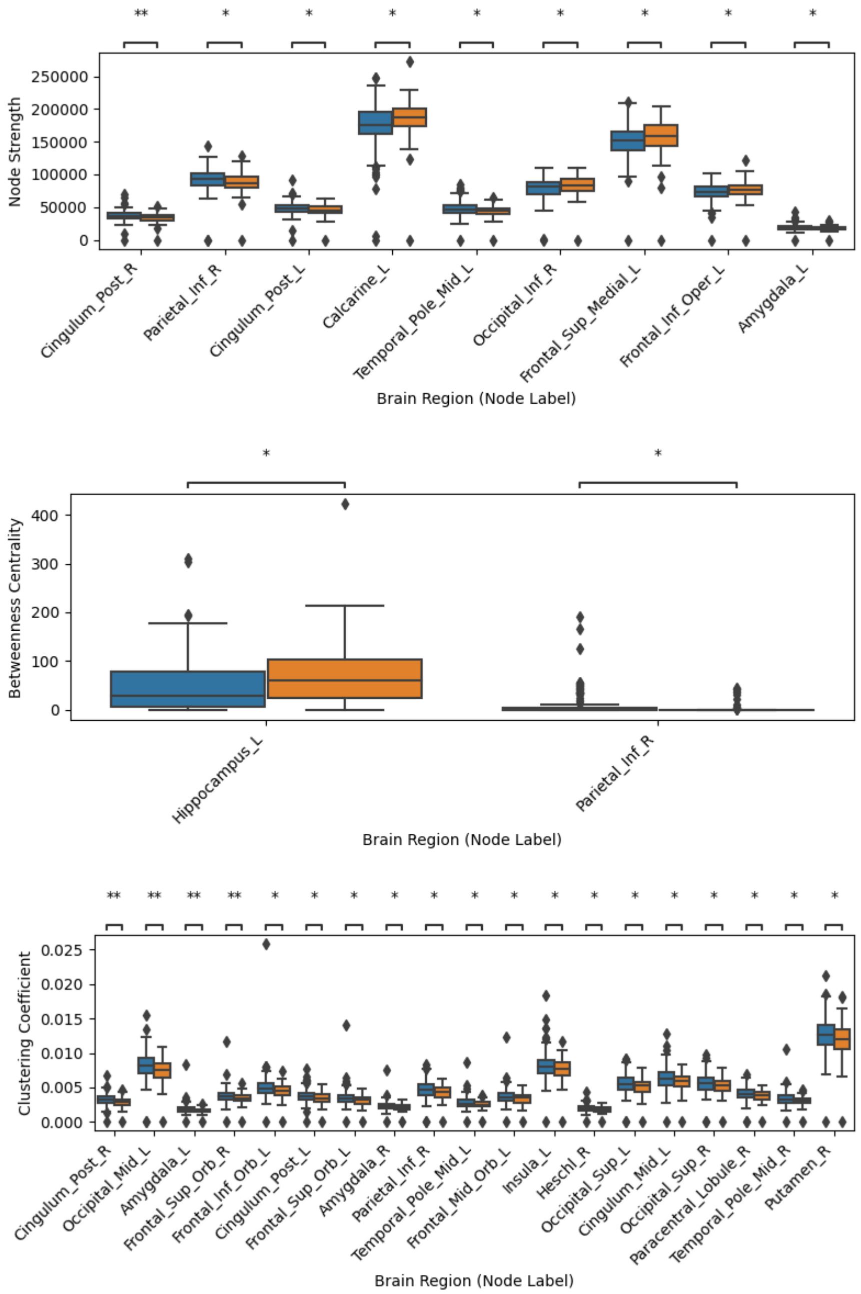

Figure A.3: Distribution of graph theory node metrics of patients (PD) in blue and healthy controls (HC) in orange for the nodes with uncorrected statistical differences for filtered connectomes. None of the differences survived correction for multiple comparisons.

A.2 Differences in connectomes estimated from fractional anisotropy

Table A.5: 10 most significant connections on unfiltered connectomes, metric: FA, hypothesis: PD>HC

Connection	abs. effect (t1)	enh. effect (t1)	uncor. P (t1)	FWE P (t1)	t val. (t1)
Caudate R↔Temporal Pole Sup L	0.0544	1538.2470	0.0046	0.0826	3.7804
Hippocampus R↔Pallidum L	0.0309	1421.1496	0.0140	0.0924	3.5309
Cingulum Post R↔Temporal Pole Mid L	0.0424	1358.7590	0.0102	0.0978	3.3782
Cingulum Ant R↔Temporal Sup L	0.0383	1309.3631	0.0092	0.1024	3.3018
Rolandic Oper R↔Temporal Inf L	0.0571	1288.2183	0.0080	0.1046	3.3464
Frontal Inf Orb L↔Pallidum L	0.0227	1256.3496	0.0164	0.1080	3.0267
Hippocampus R↔Amygdala L	0.0341	1256.3496	0.0086	0.1080	3.0055
Hippocampus L↔Pallidum R	0.0250	1242.8907	0.0174	0.1100	2.9407
Fusiform L↔Temporal Sup R	0.0371	1238.4032	0.0130	0.1102	3.1796
Cingulum Post R↔Temporal Pole Sup L	0.0345	1233.2630	0.0130	0.1112	2.9783

Table A.6: 10 most significant connections on filtered connectomes, metric: FA, hypothesis: PD>HC

Connection	abs. effect (t1)	enh. effect (t1)	uncor. P (t1)	FWE P (t1)	t val. (t1)
Caudate R↔Temporal Pole Sup L	0.0526	1433.3801	0.0052	0.0848	3.6057
Hippocampus R↔Pallidum L	0.0318	1429.0021	0.0110	0.0852	3.5539
Cingulum Post R↔Temporal Pole Mid L	0.0434	1351.2011	0.0076	0.0914	3.4409
Fusiform L↔Temporal Sup R	0.0384	1289.9169	0.0120	0.0984	3.2603
Hippocampus L↔Pallidum R	0.0280	1278.1181	0.0140	0.0996	3.2246
Cingulum Ant R↔Temporal Sup L	0.0368	1269.0371	0.0056	0.1010	3.1529
Rolandic Oper R↔Temporal Inf L	0.0566	1228.2159	0.0068	0.1050	3.2779
Cingulum Post R↔Temporal Pole Sup L	0.0346	1225.7051	0.0102	0.1052	2.9669
ParaHippocampal L↔Fusiform L	0.0227	1227.3579	0.0140	0.1052	3.0851
Hippocampus L↔Hippocampus R	0.0361	1221.3271	0.0078	0.1054	2.9857

Table A.7: 10 most significant connections on unfiltered connectomes, metric: FA, hypothesis: PD<HC

Connection	abs. effect (t2)	enh. effect (t2)	uncor. P (t2)	FWE P (t2)	t val. (t2)
Frontal Sup Medial L↔Occipital Inf R	0.0773	176.5331	0.0348	0.7914	2.6429
Frontal Inf Oper R↔Temporal Pole Sup L	0.0892	156.1327	0.0478	0.8328	2.6720
Rectus L↔Paracentral Lobule R	0.0878	147.4921	0.0472	0.8520	2.4444
Rectus L↔Calcarine R	0.0848	110.8531	0.0718	0.9202	2.1884
Cingulum Ant L↔Occipital Inf R	0.0777	89.4321	0.0842	0.9498	1.9631
Frontal Mid Orb R↔Parietal Sup L	0.0784	80.7317	0.0856	0.9630	2.1481
Cingulum Ant L↔Amygdala R	0.0647	80.3816	0.0898	0.9632	1.8514
Frontal Med Orb L↔Parietal Inf R	0.0657	77.6427	0.0884	0.9666	1.9145
Frontal Sup L↔Occipital Inf R	0.0648	71.3312	0.0944	0.9716	1.7559
Rectus L↔Occipital Inf R	0.0541	71.3312	0.0994	0.9716	1.7519

Table A.8: 10 most significant connections on filtered connectomes, metric: FA, hypothesis: PD<HC

Connection	abs. effect (t2)	enh. effect (t2)	uncor. P (t2)	FWE P (t2)	t val. (t2)
Frontal Sup Medial L↔Occipital Inf R	0.0776	175.2715	0.0404	0.7854	2.6496
Frontal Inf Oper R↔Temporal Pole Sup L	0.0893	154.8712	0.0484	0.8290	2.6693
Rectus L↔Paracentral Lobule R	0.0881	146.2306	0.0486	0.8488	2.4516
Rectus L↔Calcarine R	0.0856	109.5916	0.0676	0.9194	2.2072
Cingulum Ant L↔Occipital Inf R	0.0781	88.1705	0.0772	0.9486	1.9691
Frontal Mid Orb R↔Parietal Sup L	0.0785	80.9594	0.0818	0.9594	2.1438
Cingulum Ant L↔Amygdala R	0.0647	79.1200	0.0930	0.9630	1.8454
Frontal Sup L↔Occipital Inf R	0.0646	70.0697	0.0914	0.9720	1.7406
Rectus L↔Occipital Inf R	0.0540	70.0697	0.0968	0.9720	1.7464
Frontal Med Orb L↔Parietal Inf R	0.0651	68.6334	0.0988	0.9734	1.8966

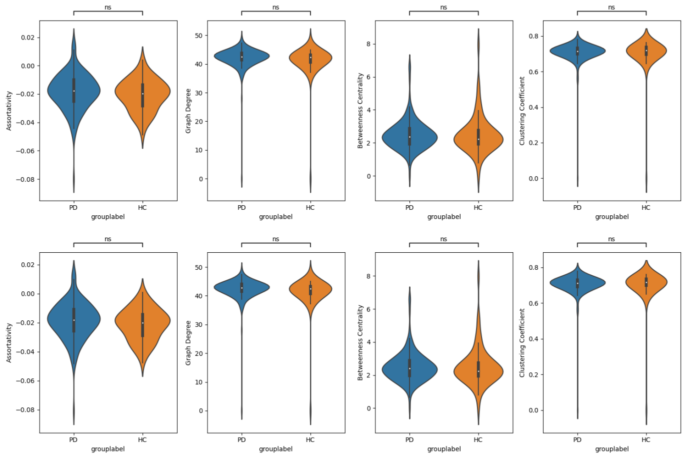

Figure A.4: Distribution of graph theory graph metrics of patients (PD) and healthy controls (HC) on unfiltered connectomes (top) and filtered connectomes (bottom).

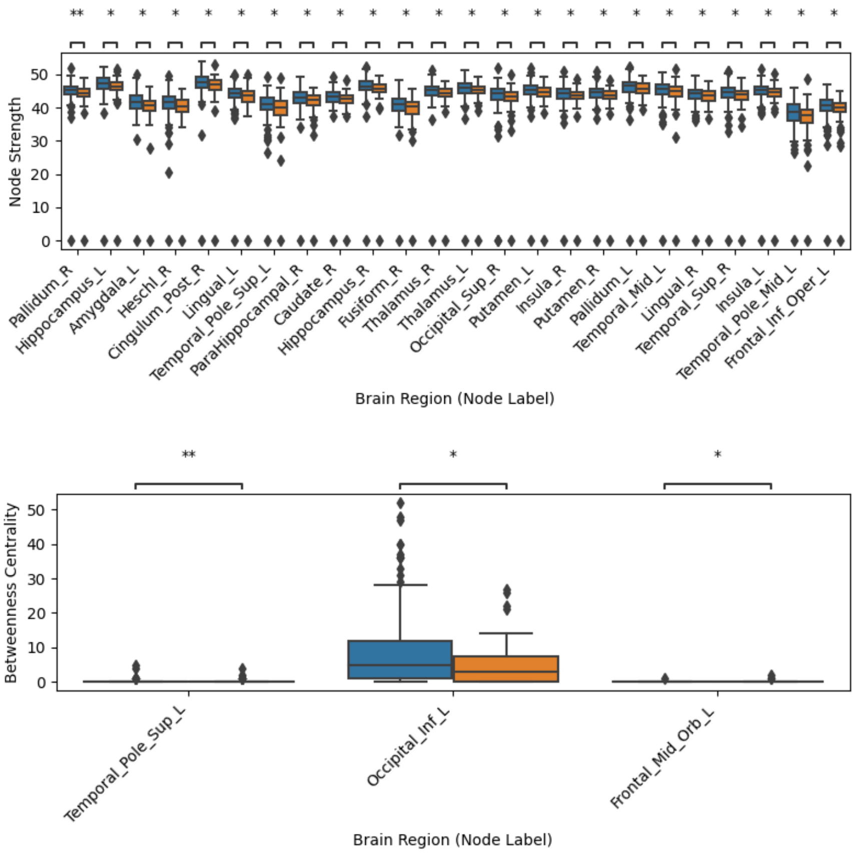

Figure A.5: Distribution of graph theory node metrics of patients (PD) in blue and healthy controls (HC) in orange for the nodes with uncorrected statistical differences for unfiltered connectomes. None of the differences survived correction for multiple comparisons.

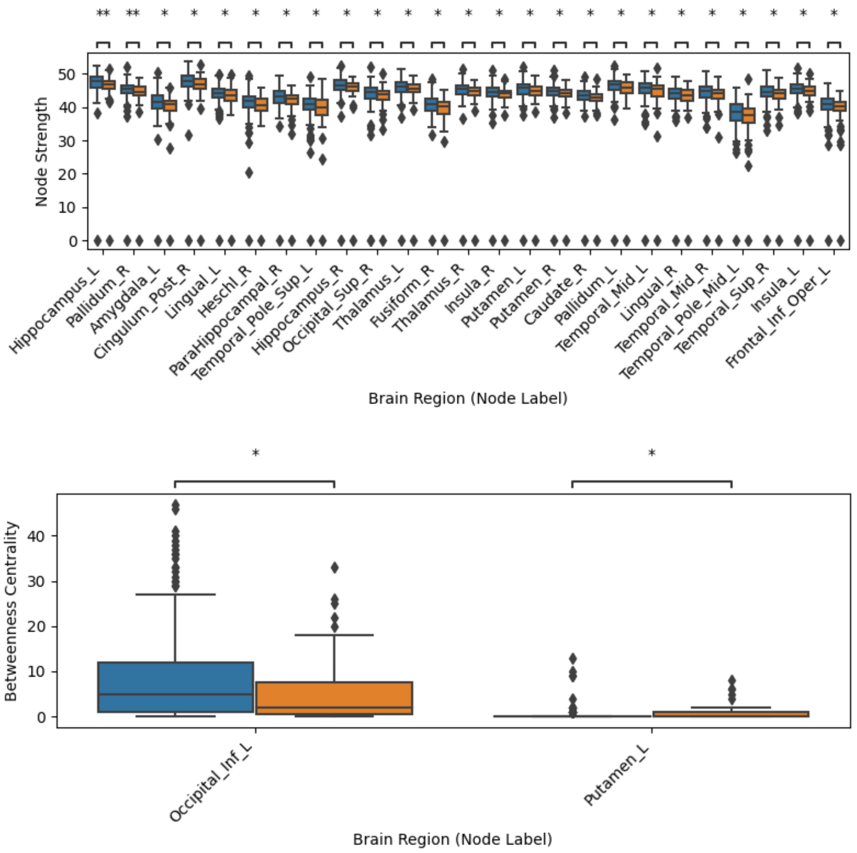

Figure A.6: Distribution of graph theory node metrics of patients (PD) in blue and healthy controls (HC) in orange for the nodes with uncorrected statistical differences for filtered connectomes. None of the differences survived correction for multiple comparisons.

A.3 Differences in connectomes estimated from axial diffusivity

Table A.9: 10 most significant connections on unfiltered connectomes, metric: AD, hypothesis: PD>HC

Connection	abs. effect (t1)	enh. effect (t1)	uncor. P (t1)	FWE P (t1)	t val. (t1)
Cingulum Ant R↔Temporal Sup L	0.0001	515.7591	0.0266	0.3380	2.9036
Caudate R↔Temporal Pole Sup L	0.0001	515.4635	0.0202	0.3384	3.0930
Cingulum Ant R↔Temporal Pole Mid L	0.0002	493.8071	0.0090	0.3580	2.7611
Cingulum Mid R↔Temporal Pole Mid L	0.0002	493.8071	0.0148	0.3580	2.7356
Cingulum Post R↔Temporal Pole Mid L	0.0001	493.8071	0.0304	0.3580	2.7631
Frontal Sup Medial R↔Temporal Sup L	0.0001	401.7307	0.0422	0.4584	2.4413
Frontal Med Orb R↔Amygdala L	0.0002	382.6431	0.0134	0.4838	2.6147
Cingulum Ant R↔Temporal Pole Sup L	0.0001	375.4145	0.0192	0.4940	2.3972
Frontal Med Orb R↔Paracentral Lobule L	0.0002	353.1941	0.0174	0.5250	2.3572
Frontal Inf Oper L↔Cingulum Post R	0.0001	348.9160	0.0414	0.5288	2.2421

Table A.10: 10 most significant connections on filtered connectomes, metric: AD, hypothesis: PD>HC

Connection	abs. effect (t1)	enh. effect (t1)	uncor. P (t1)	FWE P (t1)	t val. (t1)
Cingulum Mid R↔Temporal Pole Mid L	0.0002	513.5551	0.0178	0.3636	2.8636
Cingulum Ant R↔Temporal Sup L	0.0001	491.6031	0.0294	0.3822	2.8250
Cingulum Ant R↔Temporal Pole Mid L	0.0002	491.6031	0.0126	0.3822	2.7650
Cingulum Post R↔Temporal Pole Mid L	0.0001	491.6031	0.0336	0.3822	2.8057
Caudate R↔Temporal Pole Sup L	0.0001	486.2596	0.0258	0.3868	3.0315
Frontal Sup Medial R↔Temporal Sup L	0.0001	399.5267	0.0480	0.4730	2.4288
Frontal Med Orb R↔Amygdala L	0.0002	382.8916	0.0112	0.4936	2.6183
Cingulum Ant R↔Temporal Pole Sup L	0.0001	373.2106	0.0220	0.5048	2.4128
Frontal Med Orb R↔Paracentral Lobule L	0.0002	353.4426	0.0210	0.5282	2.3485
Olfactory L↔Fusiform R	0.0002	349.4348	0.0124	0.5332	2.4399

Table A.11: 10 most significant connections on unfiltered connectomes, metric: AD, hypothesis: PD<HC

Connection	abs. effect (t2)	enh. effect (t2)	uncor. P (t2)	FWE P (t2)	t val. (t2)
Frontal Inf Oper R↔Temporal Pole Sup L	0.0002	235.7514	0.0274	0.6934	2.9606
Rectus L↔Calcarine R	0.0003	188.9605	0.0356	0.7892	2.5557
Frontal Sup Medial L↔Occipital Inf R	0.0002	178.7120	0.0384	0.8066	2.6206
Rectus L↔Paracentral Lobule R	0.0002	159.5115	0.0428	0.8456	2.3618
Cingulum Ant L↔Amygdala R	0.0002	138.2510	0.0582	0.8840	2.3546
Rectus L↔Occipital Inf R	0.0002	117.1870	0.0678	0.9184	2.0417
Frontal Sup Orb L↔Temporal Mid R	0.0002	101.7889	0.0754	0.9414	2.1128
Frontal Med Orb L↔Parietal Inf R	0.0002	93.7889	0.0786	0.9536	1.9356
Cingulum Ant L↔Occipital Inf R	0.0002	91.3160	0.0820	0.9562	1.8287
Frontal Sup Orb L↔Parietal Inf R	0.0002	86.9299	0.0816	0.9616	1.8296

Table A.12: 10 most significant connections on filtered connectomes, metric: AD, hypothesis: PD<HC

Connection	abs. effect (t2)	enh. effect (t2)	uncor. P (t2)	FWE P (t2)	t val. (t2)
Frontal Inf Oper R↔Temporal Pole Sup L	0.0002	237.5967	0.0246	0.6754	2.9718
Frontal Sup Medial L↔Occipital Inf R	0.0002	180.5572	0.0352	0.7932	2.6137
Rectus L↔Calcarine R	0.0003	175.1808	0.0390	0.8030	2.5146
Rectus L↔Paracentral Lobule R	0.0002	161.3568	0.0460	0.8300	2.3574
Cingulum Ant L↔Amygdala R	0.0002	140.0962	0.0554	0.8704	2.3547
Rectus L↔Occipital Inf R	0.0002	119.0322	0.0696	0.9072	2.0223
Frontal Sup Orb L↔Temporal Mid R	0.0002	105.6409	0.0778	0.9324	2.0746
Frontal Med Orb L↔Parietal Inf R	0.0002	97.6409	0.0660	0.9436	1.9234
Cingulum Ant L↔Occipital Inf R	0.0002	93.1612	0.0824	0.9496	1.8150
Frontal Sup Orb L↔Parietal Inf R	0.0002	90.7819	0.0840	0.9528	1.8168

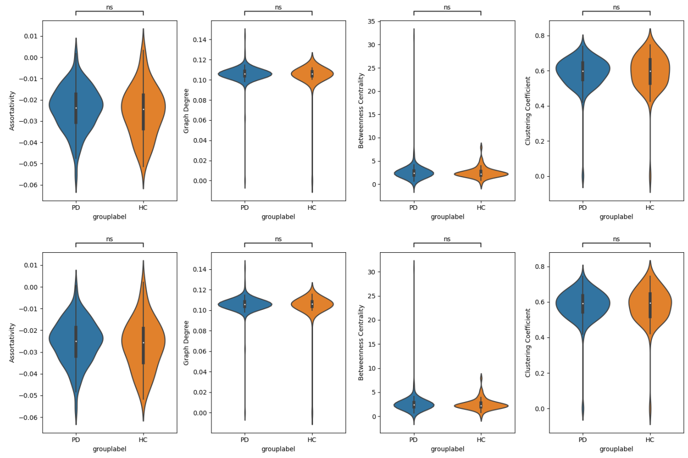

Figure A.7: Distribution of graph theory graph metrics of patients (PD) and healthy controls (HC) on unfiltered connectomes (top) and filtered connectomes (bottom).

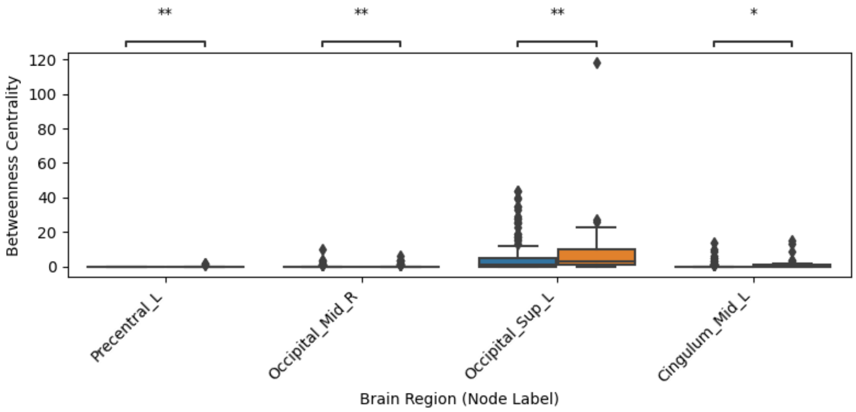

Figure A.8: Distribution of graph theory node metrics of patients (PD) in blue and healthy controls (HC) in orange for the nodes with uncorrected statistical differences for unfiltered connectomes. None of the differences survived correction for multiple comparisons.

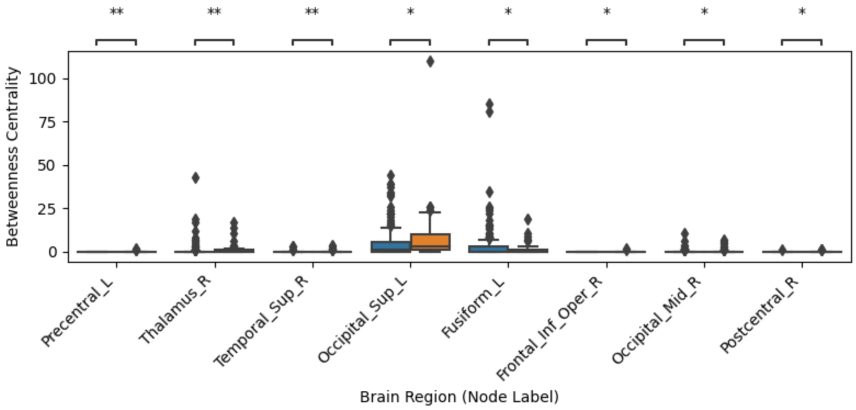

Figure A.9: Distribution of graph theory node metrics of patients (PD) in blue and healthy controls (HC) in orange for the nodes with uncorrected statistical differences for filtered connectomes. None of the differences survived correction for multiple comparisons.

A.4 Differences in connectomes estimated from radial diffusivity

Table A.13: 10 most significant connections on unfiltered connectomes, metric: RD, hypothesis: PD>HC

Connection	abs. effect (t1)	enh. effect (t1)	uncor. P (t1)	FWE P (t1)	t val. (t1)
Cingulum Mid R↔Temporal Pole Mid L	0.0001	295.2188	0.0288	0.6032	2.8392
Cingulum Ant R↔Temporal Pole Mid L	0.0001	220.3828	0.0326	0.7308	2.5099
Frontal Med Orb R↔Amygdala L	0.0001	208.6763	0.0250	0.7538	2.4826
Frontal Med Orb R↔Paracentral Lobule L	0.0001	172.0373	0.0472	0.8328	2.1651
Frontal Med Orb R↔Temporal Pole Mid L	0.0001	159.8174	0.0366	0.8572	2.0125
Frontal Sup Orb L↔Paracentral Lobule L	0.0000	144.5881	0.0704	0.8828	1.9312
Cingulum Ant R↔Temporal Sup L	0.0000	144.5881	0.0756	0.8828	1.9153
Cingulum Ant R↔Temporal Pole Sup L	0.0001	144.5881	0.0638	0.8828	2.0063
Frontal Inf Orb L↔Heschl R	0.0001	134.4091	0.0438	0.9024	2.1178
Rectus L↔Heschl L	0.0001	133.5120	0.0456	0.9048	2.0400

Table A.14: 10 most significant connections on filtered connectomes, metric: RD, hypothesis: PD>HC

Connection	abs. effect (t1)	enh. effect (t1)	uncor. P (t1)	FWE P (t1)	t val. (t1)
Cingulum Mid R↔Temporal Pole Mid L	0.0001	336.5123	0.0248	0.5468	2.9609
Cingulum Ant R↔Temporal Pole Mid L	0.0001	252.9123	0.0278	0.6840	2.5389
Frontal Med Orb R↔Amygdala L	0.0001	220.5885	0.0236	0.7464	2.4782
Frontal Med Orb R↔Paracentral Lobule L	0.0001	183.9495	0.0428	0.8140	2.2103
Frontal Med Orb R↔Temporal Pole Mid L	0.0001	171.7295	0.0378	0.8362	2.0227
Cingulum Ant R↔Temporal Sup L	0.0000	171.7295	0.0748	0.8362	2.0266
Cingulum Ant R↔Temporal Pole Sup L	0.0001	171.7295	0.0534	0.8362	2.0264
Frontal Sup Orb L↔Paracentral Lobule L	0.0000	154.3063	0.0728	0.8748	1.9631
Caudate R↔Temporal Pole Sup L	0.0000	154.3063	0.0690	0.8748	1.9839
Rectus L↔Heschl L	0.0001	153.1901	0.0506	0.8780	2.0192

Table A.15: 10 most significant connections on unfiltered connectomes, metric: RD, hypothesis: PD<HC

Connection	abs. effect (t2)	enh. effect (t2)	uncor. P (t2)	FWE P (t2)	t val. (t2)
Hippocampus R↔Pallidum L	0.0001	531.8229	0.0110	0.2884	3.5999
Frontal Inf Oper R↔Temporal Pole Sup L	0.0001	319.5403	0.0172	0.5456	3.1674
Rectus L↔Calcarine R	0.0001	250.5389	0.0252	0.6654	2.6467
Frontal Sup Orb L↔Temporal Mid R	0.0001	247.7562	0.0320	0.6712	2.7035
Cingulum Ant L↔Amygdala R	0.0001	245.0843	0.0272	0.6756	2.7316
Hippocampus L↔Pallidum R	0.0000	231.6308	0.0450	0.7026	2.5403
Frontal Sup Medial L↔Occipital Inf R	0.0001	226.6018	0.0298	0.7126	2.5837
ParaHippocampal R↔Pallidum L	0.0000	198.9319	0.0488	0.7650	2.2390
Rectus L↔Paracentral Lobule R	0.0001	191.3469	0.0382	0.7812	2.2243
Olfactory R↔Occipital Inf L	0.0001	179.4998	0.0424	0.8058	2.2154

Table A.16: 10 most significant connections on filtered connectomes, metric: RD, hypothesis: PD<HC

Connection	abs. effect (t2)	enh. effect (t2)	uncor. P (t2)	FWE P (t2)	t val. (t2)
Hippocampus R↔Pallidum L	0.0001	483.4776	0.0140	0.3330	3.5017
Frontal Inf Oper R↔Temporal Pole Sup L	0.0001	340.7433	0.0128	0.5254	3.1872
Hippocampus L↔Pallidum R	0.0000	294.2886	0.0318	0.6012	2.8906
Cingulum Ant L↔Amygdala R	0.0001	242.5812	0.0284	0.6902	2.7338
Frontal Sup Orb L↔Temporal Mid R	0.0001	236.9698	0.0286	0.6986	2.7068
Rectus L↔Calcarine R	0.0001	228.6001	0.0226	0.7124	2.5565
Frontal Sup Medial L↔Occipital Inf R	0.0001	223.4894	0.0266	0.7188	2.5658
Hippocampus L↔Hippocampus R	0.0001	182.8136	0.0516	0.8052	2.2007
ParaHippocampal R↔Pallidum L	0.0000	182.8136	0.0508	0.8052	2.1623
Precentral R↔Rectus L	0.0001	176.3361	0.0386	0.8188	2.1424

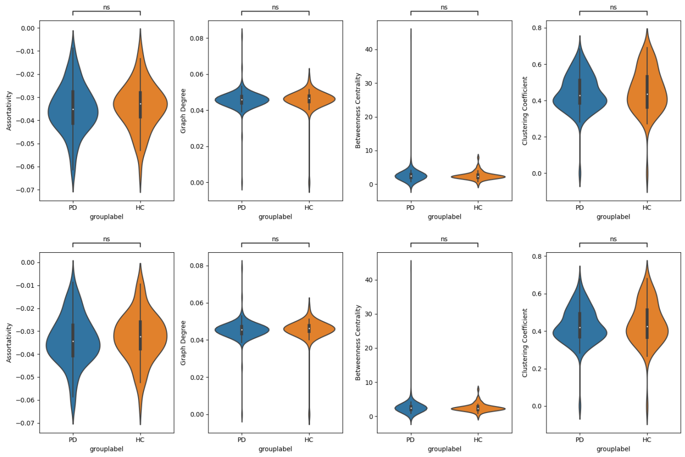

Figure A.10: Distribution of graph theory graph metrics of patients (PD) and healthy controls (HC) on unfiltered connectomes (top) and filtered connectomes (bottom).

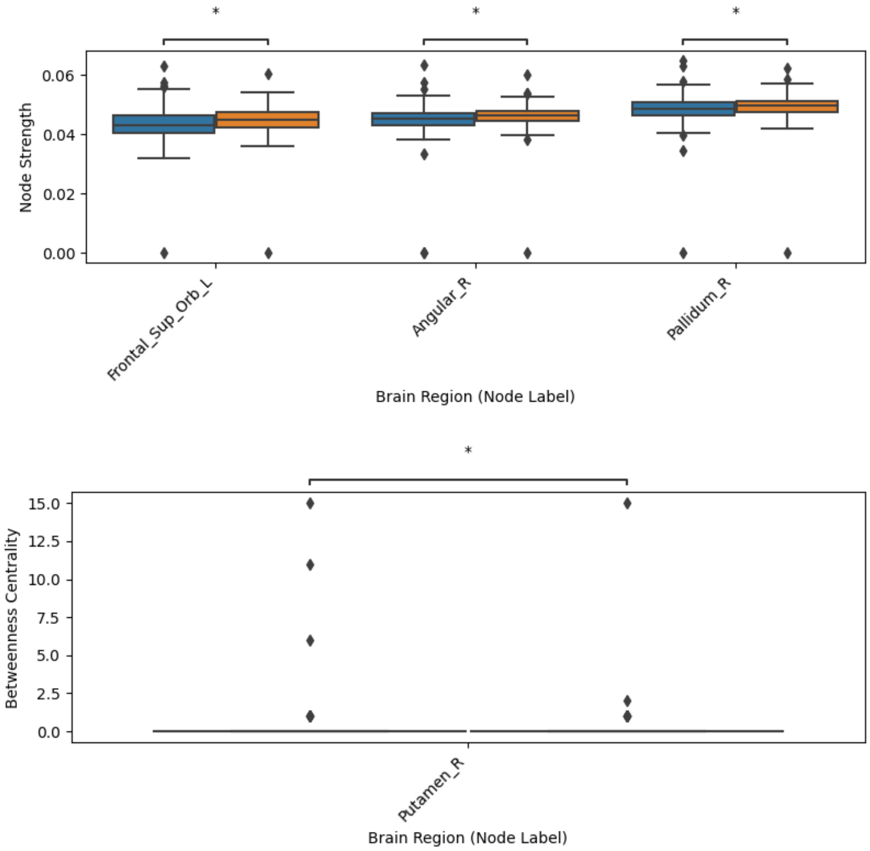

Figure A.11: Distribution of graph theory node metrics of patients (PD) in blue and healthy controls (HC) in orange for the nodes with uncorrected statistical differences for unfiltered connectomes. None of the differences survived correction for multiple comparisons.

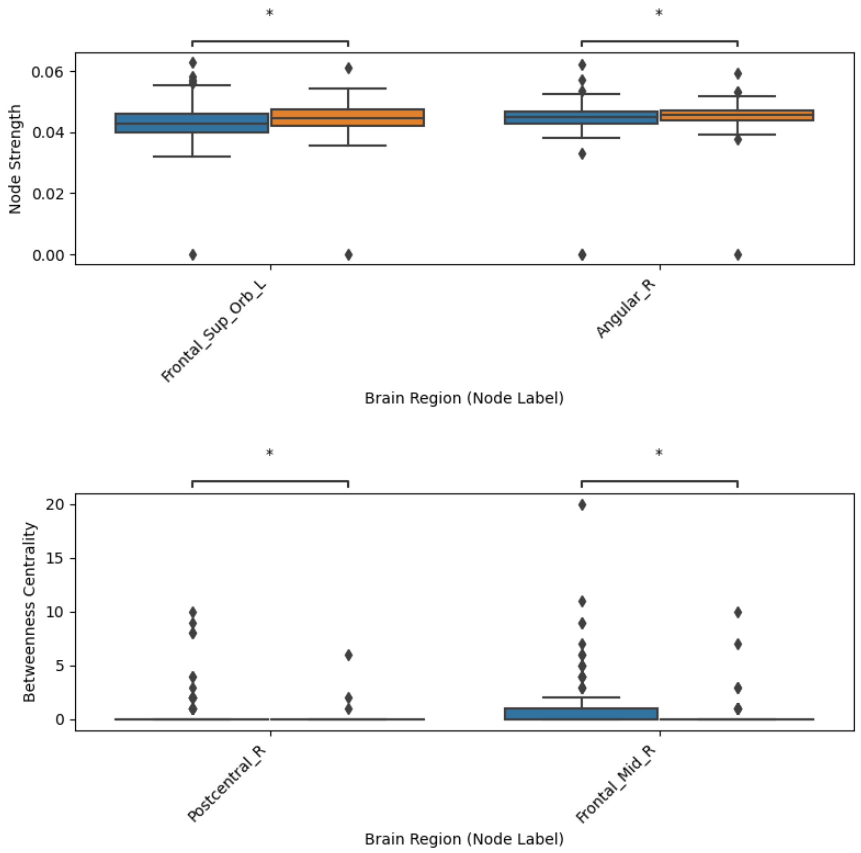

Figure A.12: Distribution of graph theory node metrics of patients (PD) in blue and healthy controls (HC) in orange for the nodes with uncorrected statistical differences for filtered connectomes. None of the differences survived correction for multiple comparisons.

A.5 Differences in connectomes estimated from mean diffusivity

Table A.17: 10 most significant connections on unfiltered connectomes, metric: MD, hypothesis: PD>HC

Connection	abs. effect (t1)	enh. effect (t1)	uncor. P (t1)	FWE P (t1)	t val. (t1)
Cingulum Mid R↔Temporal Pole Mid L	0.0001	358.9311	0.0208	0.4960	2.8027
Cingulum Ant R↔Temporal Pole Mid L	0.0001	339.2481	0.0186	0.5252	2.6524
Caudate R↔Temporal Pole Sup L	0.0001	296.7211	0.0386	0.5962	2.5466
Cingulum Ant R↔Temporal Sup L	0.0000	295.4391	0.0500	0.5976	2.4878
Frontal Med Orb R↔Amygdala L	0.0001	278.2574	0.0152	0.6254	2.5621
Cingulum Ant R↔Temporal Pole Sup L	0.0001	255.1051	0.0328	0.6656	2.2259
Cingulum Post R↔Temporal Pole Mid L	0.0000	255.1051	0.0554	0.6656	2.2596
Frontal Med Orb R↔Paracentral Lobule L	0.0001	236.6414	0.0282	0.6998	2.2783
Frontal Sup Orb L↔Paracentral Lobule L	0.0000	210.3713	0.0490	0.7508	2.0956
Supp Motor Area L↔Rectus R	0.0000	203.5441	0.0626	0.7670	2.1211

Table A.18: 10 most significant connections on filtered connectomes, metric: MD, hypothesis: PD>HC

Connection	abs. effect (t1)	enh. effect (t1)	uncor. P (t1)	FWE P (t1)	t val. (t1)
Cingulum Mid R↔Temporal Pole Mid L	0.0001	383.6529	0.0194	0.4904	2.9302
Cingulum Ant R↔Temporal Pole Mid L	0.0001	342.0179	0.0186	0.5478	2.6691
Caudate R↔Temporal Pole Sup L	0.0001	299.4909	0.0424	0.6134	2.5776
Cingulum Ant R↔Temporal Sup L	0.0000	298.2090	0.0460	0.6160	2.5053
Frontal Med Orb R↔Amygdala L	0.0001	281.0273	0.0160	0.6438	2.5621
Cingulum Ant R↔Temporal Pole Sup L	0.0001	257.8749	0.0344	0.6804	2.2452
Cingulum Post R↔Temporal Pole Mid L	0.0000	257.8749	0.0540	0.6804	2.2545
Frontal Med Orb R↔Paracentral Lobule L	0.0001	239.4113	0.0304	0.7144	2.2952
ParaHippocampal R↔Parietal Inf L	0.0000	216.9081	0.0622	0.7544	2.1130
Frontal Sup Orb L↔Paracentral Lobule L	0.0000	213.1412	0.0528	0.7624	2.0828

Table A.19: 10 most significant connections on unfiltered connectomes, metric: MD, hypothesis: PD<HC

Connection	abs. effect (t2)	enh. effect (t2)	uncor. P (t2)	FWE P (t2)	t val. (t2)
Frontal Inf Oper R↔Temporal Pole Sup L	0.0002	273.3397	0.0226	0.6192	3.0670
Hippocampus R↔Pallidum L	0.0000	205.7399	0.0476	0.7542	2.8040
Rectus L↔Calcarine R	0.0002	196.4151	0.0318	0.7748	2.6132
Frontal Sup Medial L↔Occipital Inf R	0.0001	190.0540	0.0312	0.7868	2.6081
Cingulum Ant L↔Amygdala R	0.0001	170.9020	0.0430	0.8300	2.5487
Rectus L↔Paracentral Lobule R	0.0001	154.7991	0.0490	0.8592	2.3036
Frontal Sup Orb L↔Temporal Mid R	0.0001	152.2805	0.0496	0.8648	2.4104
Rectus L↔Occipital Inf R	0.0001	128.5290	0.0586	0.9092	2.0706
Frontal Sup Orb L↔Parietal Inf R	0.0001	112.2045	0.0652	0.9326	1.9261
Frontal Med Orb L↔Lingual R	0.0001	112.2045	0.0638	0.9326	1.9359

Table A.20: 10 most significant connections on filtered connectomes, metric: MD, hypothesis: PD<HC

Connection	abs. effect (t2)	enh. effect (t2)	uncor. P (t2)	FWE P (t2)	t val. (t2)
Frontal Inf Oper R↔Temporal Pole Sup L	0.0002	283.6725	0.0202	0.5924	3.0822
Hippocampus R↔Pallidum L	0.0001	207.9720	0.0462	0.7390	2.7796
Rectus L↔Calcarine R	0.0002	200.1554	0.0302	0.7574	2.5496
Frontal Sup Medial L↔Occipital Inf R	0.0001	193.7943	0.0310	0.7688	2.5964
Cingulum Ant L↔Amygdala R	0.0001	174.6423	0.0412	0.8128	2.5505
Rectus L↔Paracentral Lobule R	0.0001	158.5394	0.0452	0.8476	2.2934
Frontal Sup Orb L↔Temporal Mid R	0.0001	152.6637	0.0514	0.8598	2.3939
Rectus L↔Occipital Inf R	0.0001	132.2693	0.0580	0.8968	2.0497
Hippocampus L↔Pallidum R	0.0000	118.4490	0.0782	0.9200	2.2110
Frontal Med Orb L↔Lingual R	0.0001	114.7792	0.0638	0.9250	1.9298

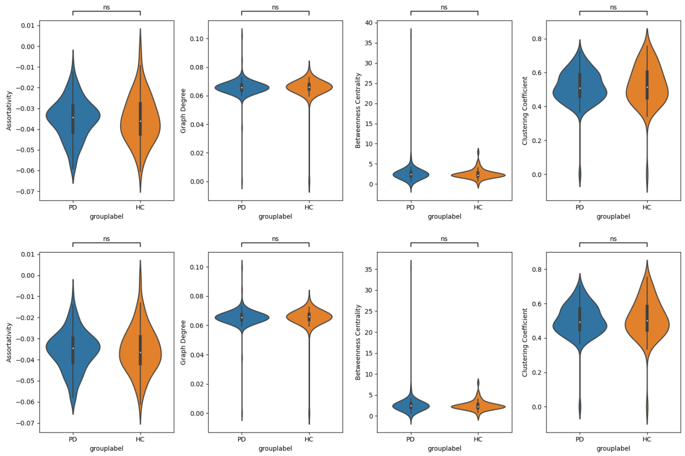

Figure A.13: Distribution of graph theory graph metrics of patients (PD) and healthy controls (HC) on unfiltered connectomes (top) and filtered connectomes (bottom).

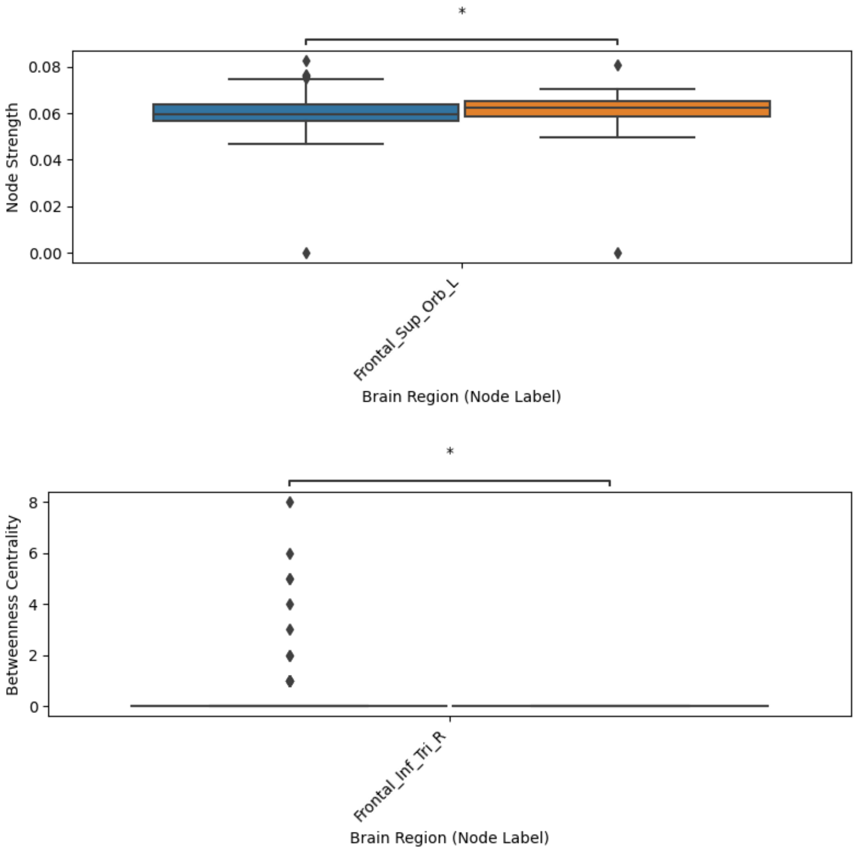

Figure A.14: Distribution of graph theory node metrics of patients (PD) in blue and healthy controls (HC) in orange for the nodes with uncorrected statistical differences for unfiltered connectomes. None of the differences survived correction for multiple comparisons.

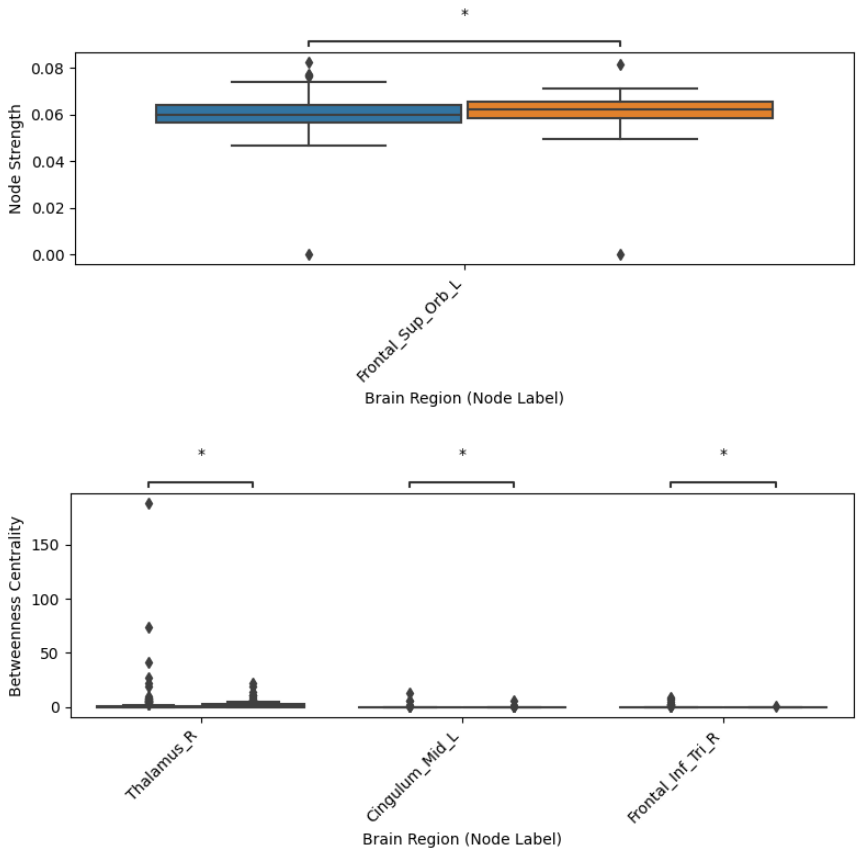

Figure A.15: Distribution of graph theory node metrics of patients (PD) in blue and healthy controls (HC) in orange for the nodes with uncorrected statistical differences for filtered connectomes. None of the differences survived correction for multiple comparisons.

A.6 Effect sizes

Table A.21: Cohen's d effect sizes for PD vs. HC group differences across graph-level metrics, comparing unfiltered and SIFT2-filtered connectomes. All effect sizes are negligible to small ($|d| < 0.3$), and the 95% confidence intervals consistently include zero, indicating no meaningful group separation. The change in effect size due to filtering (Δd) is uniformly near zero (mean $|\Delta d| = 0.027$), demonstrating that SIFT2 filtering does not alter the (already negligible) group differences. A two-factor mixed ANOVA interaction test would therefore be uninformative, as there is no substantial group effect for filtering to modulate.

Weight	Metric	d_{unfilt}	95% CI	d_{SIFT}	95% CI	Δd
SC	Assortativity	-0.348	[-0.63, -0.06]	-0.240	[-0.52, +0.04]	+0.108
	Graph Degree	-0.006	[-0.29, +0.28]	+0.003	[-0.28, +0.29]	+0.009
	Betw. Centr.	+0.016	[-0.27, +0.30]	-0.005	[-0.29, +0.28]	-0.022
	Clust. Coeff.	-0.265	[-0.55, +0.02]	-0.290	[-0.58, -0.01]	-0.025
FA	Assortativity	-0.477	[-0.76, -0.19]	-0.522	[-0.81, -0.23]	-0.046
	Graph Degree	-0.373	[-0.66, -0.09]	-0.365	[-0.65, -0.08]	+0.008
	Betw. Centr.	+0.008	[-0.28, +0.29]	+0.008	[-0.28, +0.29]	-0.000
	Clust. Coeff.	-0.142	[-0.43, +0.14]	-0.086	[-0.37, +0.20]	+0.057
MD	Assortativity	-0.033	[-0.32, +0.25]	+0.058	[-0.23, +0.34]	+0.091
	Graph Degree	-0.071	[-0.36, +0.21]	-0.073	[-0.36, +0.21]	-0.002
	Betw. Centr.	-0.025	[-0.31, +0.26]	-0.024	[-0.31, +0.26]	+0.000
	Clust. Coeff.	+0.235	[-0.05, +0.52]	+0.171	[-0.11, +0.46]	-0.064
RD	Assortativity	+0.269	[-0.02, +0.55]	+0.301	[+0.02, +0.59]	+0.032
	Graph Degree	-0.005	[-0.29, +0.28]	-0.006	[-0.29, +0.28]	-0.001
	Betw. Centr.	-0.027	[-0.31, +0.26]	-0.027	[-0.31, +0.26]	+0.000
	Clust. Coeff.	+0.293	[+0.01, +0.58]	+0.272	[-0.01, +0.56]	-0.021
AD	Assortativity	-0.344	[-0.63, -0.06]	-0.318	[-0.60, -0.03]	+0.025
	Graph Degree	-0.162	[-0.45, +0.12]	-0.163	[-0.45, +0.12]	-0.001
	Betw. Centr.	-0.022	[-0.31, +0.26]	-0.022	[-0.31, +0.26]	+0.000
	Clust. Coeff.	+0.045	[-0.24, +0.33]	+0.022	[-0.26, +0.31]	-0.023
